# Prognostic and predictive significance of nuclear HIF1α expression in locally advanced HNSCC patients treated with chemoradiation with or without nimotuzumab

**DOI:** 10.1038/s41416-020-01064-4

**Published:** 2020-09-17

**Authors:** Usha Patel, Manish Pandey, Sadhana Kannan, Tanuja A. Samant, Poonam Gera, Neha Mittal, Swapnil Rane, Asawari Patil, Vanita Noronha, Amit Joshi, Vijay M. Patil, Kumar Prabhash, Manoj B. Mahimkar

**Affiliations:** 1 Mahimkar Lab, Cancer Research Institute, Advanced Centre for Treatment, Research and Education in Cancer, Tata Memorial Centre, Navi Mumbai, Maharashtra India; 2 Homi Bhabha National Institute, Training School Complex, Anushakti Nagar, Mumbai, Maharashtra India; 3 Biostatistician, Clinical Research Secretariat, Advanced Centre for Treatment, Research and Education in Cancer, Tata Memorial Centre, Navi Mumbai, Maharashtra India; 4 Biorepository, Advanced Centre for Treatment, Research and Education in Cancer, Tata Memorial Centre, Navi Mumbai, Maharashtra India; 5 Department of Pathology, Tata Memorial Hospital, Tata Memorial Centre, Mumbai, Maharashtra India; 6 Department of Medical Oncology, Tata Memorial Hospital, Tata Memorial Centre, Mumbai, Maharashtra India

**Keywords:** Tumour biomarkers, Head and neck cancer, Tumour biomarkers, Head and neck cancer, Predictive markers

## Abstract

**Background:**

Anti-EGFR-based therapies have limited success in HNSCC patients. Predictive biomarkers are greatly needed to identify the patients likely to be benefited from these targeted therapies. Here, we present the prognostic and predictive association of biomarkers in HPV-negative locally advanced (LA) HNSCC patients.

**Methods:**

Treatment-naive tumour tissue samples of 404 patients, a subset of randomised Phase 3 trial comparing cisplatin radiation (CRT) versus nimotuzumab plus cisplatin radiation (NCRT) were analysed to evaluate the expression of HIF1α, EGFR and pEGFR by immunohistochemistry and EGFR gene copy change by FISH. Progression-free survival (PFS), locoregional control (LRC) and overall survival (OS) were estimated by Kaplan–Meier method. Hazard ratios were estimated by Cox proportional hazard models.

**Results:**

Baseline characteristics of the patients were balanced between two treatment groups (CRT vs NCRT) and were representative of the trial cohort. The median follow-up was of 39.13 months. Low HIF1α was associated with better PFS [HR (95% CI) = 0.62 (0.42–0.93)], LRC [HR (95% CI) = 0.56 (0.37–0.86)] and OS [HR (95% CI) = 0.63 (0.43–0.93)] in the CRT group. Multivariable analysis revealed HIF1α as an independent negative prognostic biomarker. For patients with high HIF1α, NCRT significantly improved the outcomes [PFS:HR (95% CI) = 0.55 (0.37–0.82), LRC:HR (95% CI) = 0.55 (0.36–0.85) and OS:HR (95% CI) = 0.54 (0.36–0.81)] compared to CRT. While in patients with low HIF1α, no difference in the clinical outcomes was observed between treatments. Interaction test suggested a predictive value of HIF1α for OS (*P* = 0.008).

**Conclusions:**

High HIF1α expression is a predictor of poor clinical response to CRT in HPV-negative LA-HNSCC patients. These patients with high HIF1α significantly benefited with the addition of nimotuzumab to CRT.

**Clinical trial registration:**

Registered with the Clinical Trial Registry of India (Trial registration identifier—CTRI/2014/09/004980).

## Background

Head and neck squamous cell carcinomas (HNSCCs) are the sixth most common cancers worldwide and comprise a major cancer burden in many regions of the world.^1^ The common risk factors associated with the disease are tobacco and/or alcohol abuse and high-risk human papilloma virus (HPV) infection.^2^ HNSCC patients are often diagnosed with locoregionally advanced (LA-HNSCC) primary disease with concurrent chemoradiation as the standard treatment of care. Anti-epidermal growth factor receptor (EGFR) therapy is the only targeted therapy approved for the treatment of LA-HNSCC patients. However, addition of anti-EGFR monoclonal antibody (mAb) to the chemoradiation regimen has largely met with limited success in these patients.^3^ Nimotuzumab (h-R3) is a humanised IgG1 mAb against EGFR shown to have low toxicity as compared to other anti-EGFR mAbs.^4,5^ Patil et al. recently reported improved progression-free survival (PFS) [hazard ratio, HR (95% CI) = 0.69 (0.53–0.89)] and locoregional control (LRC) [HR (95% CI) = 0.67 (0.50–0.89)] in unselected LA-HNSCC (> 94% HPV-negative) patients treated with nimotuzumab plus cisplatin radiation compared to the patients treated with only cisplatin radiation in a Phase 3-randomised trial conducted in India.^6^

In order to improve the clinical benefit-to-risk ratio of the given treatment, predictive biomarkers are greatly needed that can help in identifying the patients who are most likely to be benefited from the treatment. The biomarkers predictive of anti-EGFR-based therapy response are well established and are integrated into clinical care for colorectal (CRC) and non-small-cell lung cancer (NSCLC) patients.^7,8^ However, predictive biomarkers of anti-EGFR-based treatment response in HNSCCs are completely lacking, and these treatments are offered irrespective of the molecular heterogeneity across the tumours. Even though EGFR overexpression is the principal mechanism of receptor activation in HNSCCs, at present, the role of EGFR protein expression and gene copy number for predicting the response to anti-EGFR-based treatments remains equivocal as reviewed by Bossi et al.^9^ Phosphorylated EGFR dimers (pEGFR) are surrogate markers of EGFR activity; however, reports evaluating their prognostic and predictive value in LA-HNSCCs are limited. In the present study, we have analysed the expression of pEGFRY1068 and pEGFRY1173 that are among the major phosphorylation sites and are involved in the activation of important downstream pathways—PI3K-AKT and RAS-MAPK.^10^

Further, hypoxic microenvironment is a common feature of solid tumours including HNSCCs and a major contributor of radiotherapy and chemotherapy resistance.^11,12^ Hypoxia-inducible factor 1α (HIF1α) is a transcription factor that mediates adaptive responses to hypoxia by regulating numerous cellular processes such as angiogenesis, oxygen transport, glycolysis and pH control.^13,14^ HIF1α overexpression is associated with poor prognosis and resistance to chemoradiation in HNSCCs.^15^ Interestingly, several preclinical studies have demonstrated that antitumour activity of EGFR inhibitors is linked to downregulation of HIF1α expression in different cancers, including HNSCCs.^16–20^ In addition, HNSCC cell lines have been shown to be more sensitive to cetuximab under hypoxia.^21,22^ The results from these preclinical studies warrant clinical evaluation of HIF1α expression for its predictive value. In the present study, we have analysed HIF1α, EGFR and pEGFR protein expression and EGFR gene copy number in HPV-negative LA-HNSCC patients to establish a correlation between these tumour biomarkers and treatment response to cisplatin radiation and nimotuzumab plus cisplatin radiation.

## Methods

### Study design

This study included participants of a previously reported randomised Phase 3 clinical trial conducted at Tata Memorial Center, Mumbai, India (registered with the Clinical Trial Registry of India, trial registration identifier—CTRI/2014/09/004980).^6^ Briefly, 536 LA-HNSCC patients were blindly randomised 1:1 to receive radical radiotherapy (66–70 greys) with concurrent weekly cisplatin (30 mg/m^2^) (CRT arm) or the same schedule of cisplatin radiation with weekly nimotuzumab (200 mg) (NCRT arm). The primary endpoint of the trial was PFS. The present study was approved by the institutional ethics committee of Tata Memorial Center (IEC approval 50 of 2011) and was performed in accordance with the Declaration of Helsinki. This study was an independent biomarker study and not a part of the parental trial; therefore, a separate informed consent was obtained from all the participants.

### Sample collection and human papilloma virus (HPV) screening

Treatment-naive formalin-fixed paraffin-embedded (FFPE) tumour biopsy tissues and saliva samples were collected prospectively and were subjected to HPV screening. Detailed methodology for HPV screening is previously reported^23^ and is briefly described in Supplementary Methods. Out of 536 patients, biopsy tissue with adequate tumour content was available for 432 patients (80%), of which saliva samples were available for 349 patients. All 432 tumour samples were analysed for p16 protein expression by immunohistochemistry (IHC). Both saliva and tumour tissue were screened in 221 cases for HPV–DNA by PCR; for 128 cases, only saliva sample and for 54 cases only tumour tissue was analysed for HPV–DNA by PCR. A HPV-positive sample is characterised by p16-positive IHC staining and/or the presence of HPV–DNA in either tumour or saliva, along with a subsequent positive HPV RNA in situ hybridisation (RNA-ISH) test.^24^ HPV-negative tumour samples were subjected to pre-specified biomarker analysis, which was performed blinded to treatment allocation and patient’s outcomes.

### Biomarker analysis

#### Fluorescence in situ hybridisation (FISH)

EGFR gene copy number was assessed by FISH using EGFR/CEP7 FISH probe (Abbott Vysis, CA, USA). A detailed protocol is provided in Supplementary Methods. FISH signals were counted in at least 100 tumour cells under ×63 magnification. EGFR gene copy status was classified into five categories, depending on the percentage of tumour cells showing different copies of EGFR gene locus and centromere as disomy (≤2 copies in >90% of cells), trisomy (3 copies in ≥10% of cells or ≥4 copies in <10% of cells), low polysomy (≥4 copies in 10–40% of cells), high polysomy (≥4 copies in ≥40% of cells) and gene amplification (ratio of the EGFR gene to chromosome 7 of ≥2 or ≥15 copies of EGFR per cell in ≥10% of cells). On the basis of EGFR gene copy status, patients were grouped as FISH-negative (disomy, trisomy and low polysomy) or FISH-positive (high polysomy and/or EGFR gene amplification).^25^

#### Immunohistochemistry

Protein expression of HIF1α, EGFR, pEGFRY1068 and pEGFRY1173 was analysed by IHC using VECTASTATIN® Elite® ABC kit (Vector Laboratories, CA, USA). A detailed protocol is provided in Supplementary Methods, and details of primary antibodies and positive controls are tabulated in Supplementary Table 1. IHC staining was evaluated semi-quantitatively by the pathologists who were blinded to treatment and patient’s outcomes. Expression of HIF1α (nuclear), EGFR (membrane and cytoplasmic), pEGFRY1068 (membrane) and pEGFRY1173 (membrane) was assessed by deriving the H-score (scale: 1–300) using the formula H-score = Σ_*Pi*_ (*i* + 1), where _*Pi*_ is the percentage (0–100%) of stained tumour cells at each intensity and *i* is the intensity: *i* = 1 (weak), 2 (moderate) and 3 (strong).^9,15^ Biomarkers were analysed as dichotomised variables. Due to unavailability of consensus regarding H-score cut point to be used for dichotomisation of these biomarkers, the respective median H-score values were used for HIF1α (H-score = 90) and EGFR (membrane, H-score = 100; cytoplasm, H-score = 140).^26,27^ For pEGFRY1068 and pEGFRY1173, patients with H-score = 0 were categorised as negative and patients with H-score > 0 were positive. IHC staining of HIF1α was independently evaluated by a second pathologist.

### Statistical analysis

Categorical data are presented as frequency and percentage; continuous data are expressed by median and range or interquartile range (IQR). Bivariate association between different biomarkers and between biomarkers and clinicopathological parameters was analysed by Pearson’s χ^2^ test. PFS, LRC and overall survival (OS) were as defined earlier^6^ and were estimated using Kaplan–Meier method and compared by log-rank tests. Cox proportional hazard models were used to derive hazard ratios (HR) and 95% confidence intervals (CI). The definition used for prognostic and predictive biomarkers was as proposed by Clark et al.^28^ For assessing the prognostic significance of each biomarker, only patients from the CRT arm were included in the analysis. In addition, however, we have also studied the association of biomarkers with clinical outcomes in the NCRT arm. Univariate Cox models were applied to select the most promising biomarkers (threshold *P* < 0.20). A multivariate Cox model using backward likelihood ratio (LR) method was then applied to adjust for potential confounders (clinical characteristics associated with PFS, LRC or OS at *P* < 0.20). Reported HRs (95% CI) are for low or negative biomarker expression relative to high or positive biomarker expression. For assessing the predictive significance of each biomarker, all patients with biomarker data, irrespective of the treatment group, were included in the analyses. Cox models were fit, which included treatments (NCRT vs CRT), biomarker status (low/negative vs high/positive) and the interaction between treatment effect and biomarker status.^28,29^ Internal validation of prognostic and predictive models was achieved by bootstrap-resampling method (1000 samples). Concordance indexes (c indexes) were also calculated.

Agreement between IHC scoring of HIF1α by two pathologists (SR and NM) was assessed using the Bland–Altman plot, and the concordance correlation coefficient was derived.^30,31^ Scoring of SR was used for analysis after obtaining consensus in the cases with H-score difference of >100, which were jointly reviewed by both the pathologists. Statistical analyses were performed using IBM SPSS software version 21 (SPSS Inc., IL, USA); STATA version 14 (StataCorp, TX, USA) was used for the bootstrap procedure and for generating forest plots; all reported *P* values are two-sided and *P* value of 0.05 or less was considered statistically significant. The study followed the REMARK guidelines for reporting.^32,33^

## Results

### Patients and HPV screening

**Fig. 1:**
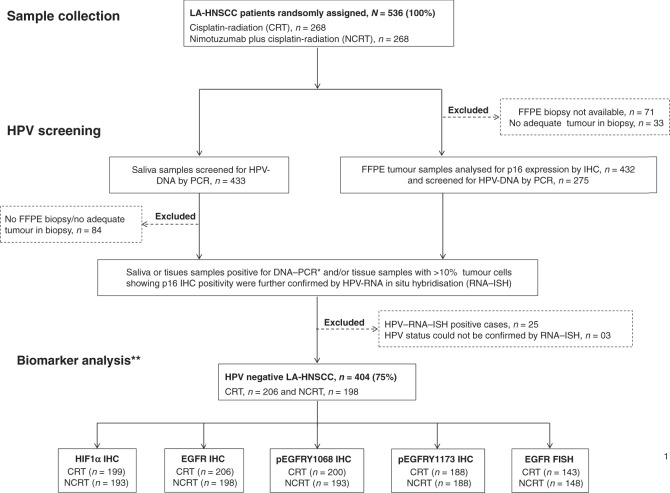
Flow diagram of the study. (*) Both saliva and tumour tissue were screened in 221 cases for HPV–DNA by PCR; for 128 cases, only saliva sample and for 54 cases only tumour tissue was analysed for HPV–DNA by PCR. (**) Biomarker groups differed in sample size due to limited availability of biopsy tumour tissue; LA-HNSCC   locally advanced HNSCC, HPV   human papilloma virus, FISH   fluorescence in situ hybridisation.

Out of 432 cases screened for HPV, 25 (5.8%) cases showed the presence of transcriptionally active high-risk HPV (Supplementary Fig. 1) and the results were inconclusive in 3 (0.7%) cases. We excluded these 28 cases and carried out biomarker analysis in the remaining 404 HPV-negative cases out of which 206 received CRT and 198 received NCRT treatment. The workflow of the study is outlined in Fig. 1. Baseline characteristics of the patients were balanced between the two treatment groups, and were representative of the total trial population (Table 1). Kaplan–Meier plots showing the treatment outcomes in the biomarker subgroup (*n* = 404) are provided in Supplementary Fig. 2. A total of 241 patients (45%) had died at the time of analysis, and the median follow-up of patients still alive was 39.13 months; 4-year survival rates are reported.

**Table 1:** Demographics and baseline characteristics of HNSCC patients enrolled in a randomised clinical trial, CTRI/2014/09/004980, Tata Memorial Hospital, India.

Characteristics	Trial population (*N* = 536)		Biomarker subgroup (*N* = 404)		
	CRT (*n* = 268)	NCRT (*n* = 268)	CRT (*n* = 206)	NCRT (*n* = 198)	*P* value
*Age (years)*					
Median and range	54 (26–77)	55 (20–73)	54 (28–77)	56 (23–73)	
40 or below	26 (9.7)	30 (11.2)	16 (7.8)	19 (9.6)	0.217
>40 and <60	165 (61.6)	156 (58.2)	132 (64.1)	110 (55.6)	
60 and above	77 (28.7)	82 (30.6)	58 (28.1)	69 (34.8)	
*Gender*					
Male	231 (86.2)	226 (84.3)	181 (88.3)	171 (86.4)	0.653
Female	37 (13.8)	42 (15.7)	25 (11.7)	27 (13.6)	
*ECOG PS*					
0	58 (21.6)	60 (22.4)	47 (22.8)	44 (22.2)	0.887
1–2	210 (78.4)	208 (77.6)	159 (77.2)	154 (77.8)	
*Site of tumour*					
Hypopharynx	47 (17.5)	62 (23.1)	42 (20.4)	49 (24.7)	0.174
Larynx	83 (31)	72 (26.9)	66 (32)	49 (24.7)	
Oral cavity	3 (1.1)	0 (0)	2 (1)	0 (0)	
Oropharynx	135 (50.4)	134 (50)	96 (46.6)	100 (50.5)	
*Clinical stage*^a^					
II	5 (1.9)	4 (1.5)	0 (0)	0 (0)	0.158
III	77 (28.7)	65 (24.3)	58 (28.2)	40 (20.2)	
IVA	80 (29.9)	81 (30.2)	57 (27.7)	65 (32.8)	
IVB	106 (39.6)	118 (44.0)	91 (44.2)	93 (47.0)	
*T stage*^a^					
T1–T2	56 (20.9)	41 (15.3)	41 (19.9)	34 (17.2)	0.48
T3–T4	212 (79.1)	227 (84.7)	165 (80.1)	164 (82.8)	
*N stage*^a^					
N0–N1	107 (39.9)	92 (34.3)	80 (38.8)	64 (32.3)	0.172
N2–N3	161 (60.1)	176 (65.7)	126 (61.2)	134 (67.7)	
*Tobacco and alcohol habits*					
No habits	27 (10.1)	30 (11.2)	14 (6.8)	16 (8.1)	0.513
Exclusive chewer	44 (16.4)	48 (17.9)	36 (17.5)	40 (20.2)	
Exclusive smoker^b^	50 (18.6)	49 (18.3)	37 (18)	33 (16.7)	
Exclusive drinker	3 (1.1)	8 (3)	1 (0.5)	4 (2)	
Mixed habits^c^	139 (51.9)	121 (45.1)	114 (55.3)	98 (49.5)	
No information	5 (1.9)	12 (4.5)	4 (1.9)	7 (3.5)	

*CRT* cisplatin radiation, *NCRT* nimotuzumab plus cisplatin radiation, *ECOG* Eastern Cooperative Oncology Group.

Data are the number (%) unless otherwise indicated. ^a^According to AJCC-UICC system (8th edition); ^b^bidi or cigarette smoking; ^c^tobacco chewing along with bidi/cigarette smoking and/or alcohol drinking; *P* value, Pearson Chi-square test.

### Expression of biomarkers

Expression of total EGFR, pEGFRY1068, pEGFRY1173 and HIF1α was assessed by IHC staining, and EGFR gene copy status was evaluated by FISH (Supplementary Figs. 3 and 4). The frequency distribution of protein biomarker expression (Supplementary Fig. 5) and EGFR–FISH status (Supplementary Table 2) was comparable between two treatment groups. Overall, the expression of pEGFRY1068 and pEGFRY1173 showed a skewed distribution as >80% and >70% of the cases respectively were negative (H-score = 0) in both treatment groups. We did not find any strong correlation among the studied biomarkers (Supplementary Table 3). However, moderate correlation was observed between membrane and cytoplasmic EGFR (*R* = 0.40), as well as between pEGFRY1068 and pEGFRY1173 (*R* = 0.57). Both membrane and cytoplasmic EGFR expression showed weak correlation with pEGFR dimers. A weak correlation was also observed between HIF1α and membrane EGFR expression (*R* = 0.15). No statistically significant association was observed between biomarkers and patient’s clinical characteristics, except for the cytoplasmic EGFR that was associated with disease stage (*P* = 0.027, Supplementary Table 4).

### Prognostic significance

**Fig. 2:**
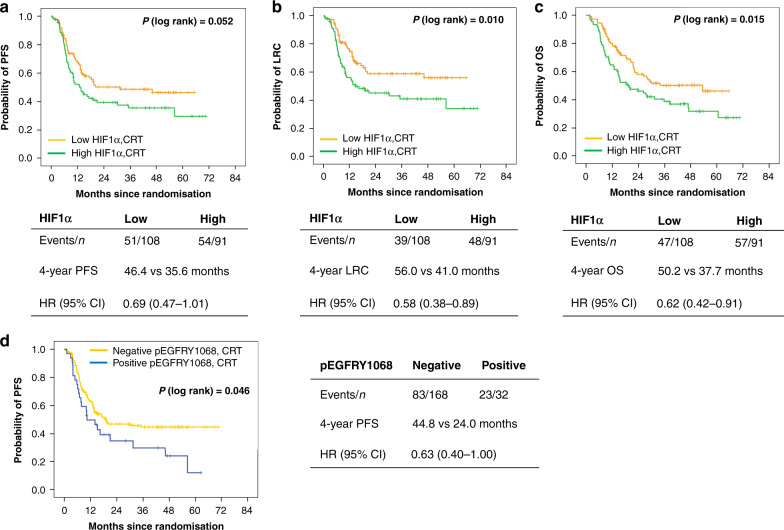
Prognostic value of HIF1α and pEGFRY1068. Kaplan–Meier curves showing PFS (**a**), LRC (**b**), OS (**c**) according to HIF1α expression status and PFS (**d**) according to pEGFRY1068 status in the CRT group; HR   hazard ratio, CI   confidence interval, PFS progression-free survival, LRC locoregional control, OS overall survival, CRT cisplatin radiation.

Univariate Cox regression analysis performed at different HIF1α H-score cut points indicated that low HIF1α expression was numerically associated with better PFS, LRC and OS in the CRT group (Supplementary Table 5A). Unadjusted analyses using the median cut point showed that the low HIF1α expression was significantly associated with better LRC [HR (95% CI) = 0.58 (0.38–0.89), *P* = 0.011] as well as OS [HR (95% CI) = 0.62 (0.42–0.91), *P* = 0.016], and showed a trend towards improved PFS [HR (95% CI) = 0.69 (0.47–1.01), *P* = 0.053, Fig. 2a–c]. EGFR expression (membrane or cytoplasmic) studied at different cut points including the median did not associate with PFS, LRC or OS in the CRT group (Supplementary Table 5B, C). Patients with negative pEGFRY1068 status showed improved PFS compared to patients with positive pEGFRY1068 [HR (95% CI) = 0.63 (0.40–1.0), *P* = 0.048, Fig. 2d]; similar difference was not observed in LRC or OS (Supplementary Table 6). pEGFRY1173 and EGFR–FISH status did not show any association with the clinical outcomes (Supplementary Table 6). Multivariable analysis adjusted for confounding variables with a univariate *P* < 0.20 in Supplementary Table 7 (age, clinical stage and site of tumour) identified low HIF1α as an independent prognostic biomarker for improved PFS [HR (95% CI) = 0.62 (0.42–0.93), *P* = 0.020], LRC [HR (95% CI) = 0.56 (0.37–0.86), *P* = 0.007] and OS [HR (95% CI) = 0.63 (0.43–0.93), *P* = 0.019] in the CRT group (Table 2). Further validation by bootstrap-resampling method confirmed the prognostic effect of HIF1α; low HIF1α was significantly associated with better outcomes [PFS: HR (95% CI) = 0.64 (0.43–0.96), *P* = 0.031, c index (95% CI) = 0.61 (0.55–0.66); LRC: HR (95% CI) = 0.58 (0.37–0.89), *P* = 0.012, c index (95% CI) = 0.62 (0.56–0.68); OS: HR (95% CI) = 0.63 (0.42–0.94), *P* = 0.025, c index (95% CI) = 0.60 (0.54–0.65)] in the CRT group. We did not find significant association between any of the studied biomarkers and clinical outcomes among patients in the NCRT group (Supplementary Table 8).

**Table 2:** Prognostic significance of clinical parameters and biomarkers in the cisplatin-radiation group.

Variables	Univariate Cox analysis		Multivariable Cox analysis*	
	HR (95% CI)	*P* value	HR (95% CI)	*P* value
*Progression-free survival (PFS)*				
Age (below 60 vs 60 & above)	1.46 (0.94–2.28)	0.092	1.56 (0.97–2.52)	0.066
^#^Clinical stage (III vs IV)	0.48 (0.30–0.78)	0.003	0.41 (0.24–0.71)	0.001
Site of tumour (oropharynx vs others)	1.74 (1.19–2.56)	0.004	–	–
pEGFRY1068 (negative vs positive)	0.63 (0.40–1.0)	0.048	–	–
pEGFRY1173 (negative vs positive)	0.74 (0.48–1.14)	0.17	–	–
HIF1α (low vs high)	0.69 (0.47–1.01)	0.053	0.62 (0.42–0.93)	0.020
*Locoregional control (LRC)*				
Age (below 60 vs 60 & above)	1.49 (0.91–2.43)	0.111	1.57 (0.96–2.56)	0.075
^#^Clinical stage (III vs IV)	0.43 (0.25–0.75)	0.003	0.39 (0.22–0.67)	0.001
Site of tumour (oropharynx vs others)	1.58 (1.05–2.40)	0.030	–	–
HIF1α (low vs high)	0.58 (0.38–0.89)	0.011	0.56 (0.37–0.86)	0.007
*Overall survival (OS)*				
Age (below 60 vs 60 & above)	1.59 (1.0–2.53)	0.049	1.65 (1.10–2.38)	0.036
^#^Clinical stage (III vs IV)	0.64 (0.40–1.00)	0.051	–	–
Site of tumour (oropharynx vs others)	1.62 (1.10–2.37)	0.014	1.62 (1.10–2.38)	0.015
HIF1α (low vs high)	0.62 (0.42–0.91)	0.016	0.63 (0.43–0.93)	0.019

*HR* hazard ratio, *CI* confidence interval, (*–*) data not available.

*A multivariate Cox model using backward likelihood ratio method was applied to adjust for potential confounders (clinical characteristics associated with PFS, LRC or OS at *P* < 0.20 in univariate analysis). ^#^According to AJCC-UICC system (8th edition).

### Predictive significance

**Fig. 3:**
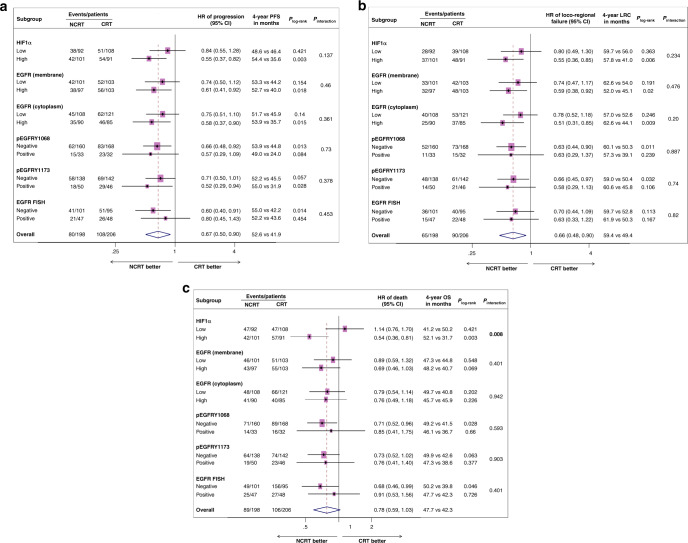
Forest plots showing predictive association of the studied biomarkers. PFS (**a**), LRC (**b**) and OS (**c**). The interaction *P* value is based on a two-sided test of interaction between treatment and biomarker expression status in the Cox proportional hazard model. A hazard ratio (HR) of <1 indicates a benefit with the addition of nimotuzumab. CI confidence interval, PFS progression-free survival, LRC locoregional control, OS overall survival.

**Fig. 4:**
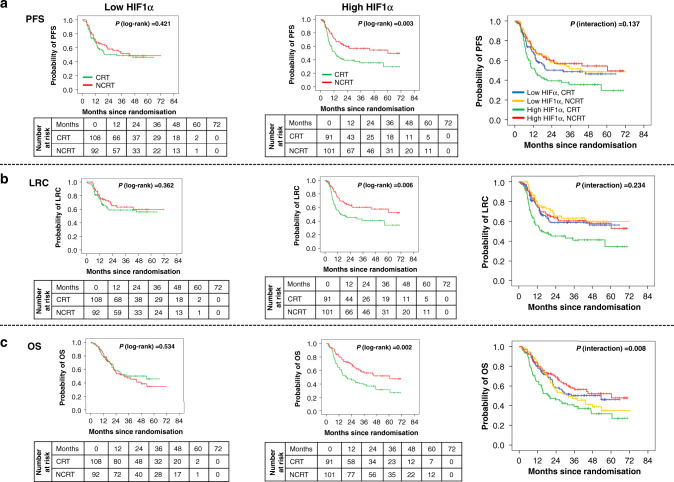
HIF1α showing qualitative interaction. Kaplan–Meier curves showing, PFS (**a**), LRC (**b**) and OS (**c**) for LA-HNSCC patients according to HIF1α expression status and treatment group. PFS progression-free survival, LRC locoregional control, OS overall survival.

Interestingly, univariate Cox analysis showed that patients with high HIF1α had significantly improved PFS [HR (95% CI) = 0.55 (0.37–0.82), *P* = 0.003], LRC [HR (95% CI) = 0.55 (0.36–0.85), *P* = 0.006] and OS [HR (95% CI) = 0.54 (0.36–0.81), *P* = 0.003] with NCRT compared to CRT. Similar benefits in PFS, LRC or OS were not observed in low HIF1α-expressing subgroups with NCRT versus CRT (Figs. 3a–c and 4a–c). A statistically significant qualitative interaction was observed between treatment and HIF1α status for OS [*P* = 0.008] but not for PFS [*P* = 0.137] or LRC [*P* = 0.234]. The predictive value of HIF1α was further validated by bootstrap-resampling method [OS: *P* (interaction)= 0.007, c index (95% CI) = 0.57 (0.52–0.61)]; forest plots representing the interaction between treatments and HIF1α status for PFS, LRC and OS are provided in Supplementary Fig. 6. In addition, analysis carried out at different cut points revealed that overall high HIF1α expression was associated with better outcomes in NCRT as compared to CRT, with minimum-interaction *P* value observed at the median cut point (Supplementary Table 9). Immunostaining of HIF1α was independently evaluated by a second pathologist (NM); a good agreement was observed between scoring of two pathologists (S.R. and N.M.) as shown by Bland–Altman plot (Supplementary Fig. 7) with concordance correlation coefficient (95% CI) of 0.89 (0.87–0.91).^30,31^

**Fig. 5:**
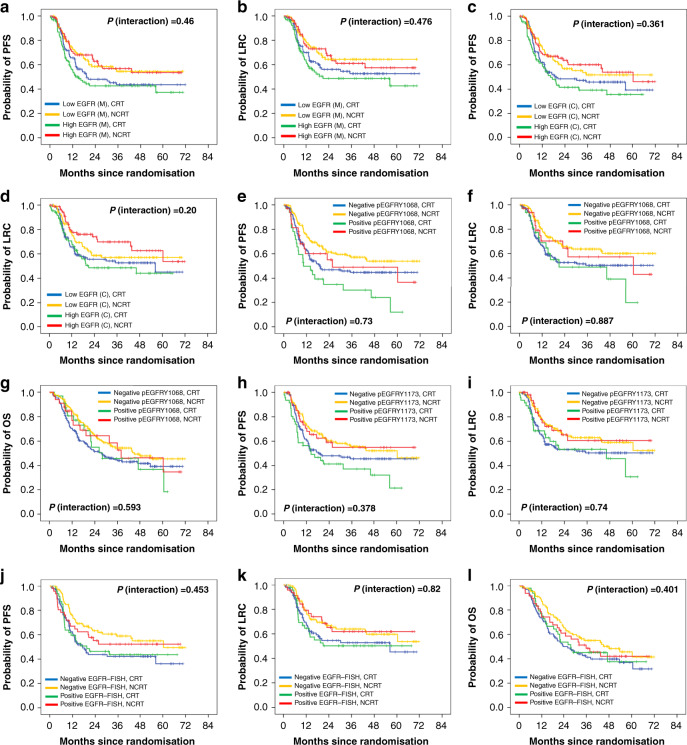
Kaplan–Meier curves stratified by biomarker status and treatment. PFS (**a**) and LRC (**b**) according to EGFR (membrane) expression status and treatment group; PFS (**c**) and LRC (**d**) according to EGFR (cytoplasmic) expression status and treatment group; PFS (**e**), LRC (**f**) and OS (**g**) according to pEGFRY1068 expression status and treatment group; PFS (**h**) and LRC (**i**) according to pEGFRY1173 expression status and treatment group; PFS (**j**), LRC (**k**) and OS (**l**) according to EGFR–FISH status and treatment group. PFS progression-free survival, LRC locoregional control, OS overall survival.

We next analysed the predictive impact of EGFR-based biomarkers. Univariate Cox analysis showed that PFS [HR (95% CI) = 0.61 (0.41–0.92), *P* = 0.02] and LRC [HR (95% CI) = 0.59 (0.38–0.92), *P* = 0.021] were significantly improved in the patients expressing high-membrane EGFR with NCRT versus CRT, while the difference in OS was not statistically significant [HR (95% CI) = 0.69 (0.46–1.03), *P* = 0.071]. Improvement in PFS, LRC or OS with NCRT versus CRT was not observed in patients with low-membrane EGFR expression (Figs. 3a–c and 5a, b). Similar associations were also observed between cytoplasmic EGFR and treatment effect. Patients with high cytoplasmic EGFR expression had statistically significant better PFS [HR (95% CI) = 0.58 (0.37–0.90), *P* = 0.016] and LRC [HR (95% CI) = 0.51 (0.31–0.85), *P* = 0.01] but not OS [HR (95% CI) = 0.76 (0.49–1.18), *P* = 0.228] with NCRT versus CRT (Figs. 3a–c and 5c, d). Similar benefits in PFS, LRC or OS were not observed in the patients with low cytoplasmic EGFR with NCRT compared to CRT (Figs. 3a–c and 5c, d). We did not find any significant interaction between treatment and EGFR (membrane or cytoplasmic) expression status at any of the studied cut points for PFS, LRC or OS (Supplementary Table 10A, B).

Further, NCRT significantly improved the outcomes in patients with negative pEGFRY1068 status [PFS: HR (95% CI) = 0.66 (0.48–0.92), *P* = 0.014; LRC: HR (95% CI) = 0.63 (0.44–0.90), *P* = 0.012; OS: HR (95% CI) = 0.71 (0.52–0.96), *P* = 0.029], but offered no benefit in patients with positive pEGFRY1068 (Figs. 3a–c and 5e–g). We did not find any interaction between treatment and pEGFRY1068 status for any of the studied endpoints. We observed better LRC in patients with negative pEGFRY1173 with NCRT versus CRT [HR (95% CI) = 0.66 (0.45–0.97), *P* = 0.034]; however, significant improvements in PFS were observed in patients with positive pEGFRY1173 with NCRT [HR (95% CI) = 0.52 (0.29–0.94), *P* = 0.031]. Interaction between treatments and pEGFRY1173 status was nonsignificant for all the studied endpoints (Figs. 3a–c and 5h, i). It should be noted that in this study, subgroups with positive pEGFR expression were small in number; therefore, these results need further validation. PFS [HR (95% CI) = 0.60 (0.40–0.91), *P* = 0.015] and OS [HR (95% CI) = 0.68 (0.46–0.99), *P* = 0.047] were significantly improved with NCRT in patients with EGFR–FISH-negative status; however, difference in LRC between treatments was not significant [HR (95% CI) = 0.63 (0.33–1.22), *P* = 0.167, Figs. 3a–c and 5j–l]. Similar benefits in PFS, LRC or OS were not observed in FISH-positive patients; the interaction between treatment and EGFR–FISH status was found to be nonsignificant (Figs. 3a–c and 5j–l). Taken together, these results suggest that the treatment effect of NCRT is independent of EGFR protein or gene copy status in these patients.

Furthermore, we carried out combined predictive analysis of HIF1α and membrane EGFR (Supplementary Table 11). Patients with high expression of both HIF1α and membrane EGFR had better PFS [HR (95% CI) = 0.57 (0.33–0.98), *P* = 0.04], LRC [HR (95% CI) = 0.54 (0.30–0.96), *P* = 0.036] and OS [HR (95% CI) = 0.51 (0.29–0.87), *P* = 0.013] with NCRT compared to CRT. Similar improvement in PFS was also observed in patients with high HIF1α and low-membrane EGFR with NCRT versus CRT [HR (95% CI) = 0.52 (0.28–0.96), *P* = 0.036]; however, the improvement in LRC [HR (95% CI) = 0.57 (0.29–1.09), *P* = 0.088] and OS [HR (95% CI) = 0.60 (0.33–1.10), *P* = 0.097] did not reach statistical significance. In the remaining two subgroups that include a subgroup with low expression of both biomarkers and another with low HIF1α along with high EGFR, we did not find any significant difference in PFS, LRC or OS between the treatment groups. Overall, combined analysis of HIF1α–EGFR did not show any additional predictive value over HIF1α alone.

## Discussion

Prognostic biomarkers are extensively studied in HNSCCs, but they have limited utility in patients’ treatment decisions. While the identification of predictive biomarkers is a pressing need to enable selection of patients for a specific treatment. In the present study, we have evaluated prognostic and predictive significance of HIF1α, EGFR, pEGFR protein expression and EGFR gene copy number in HPV-negative LA-HNSCC patients treated either with CRT or NCRT in a Phase 3-randomised study. Here we have shown high HIF1α as an independent negative prognostic factor for PFS, LRC and OS in patients treated with CRT. Interestingly, addition of nimotuzumab to CRT significantly improved the clinical outcomes in patients expressing high HIF1α, with 45% less risk of progression, 45% less risk of locoregional failure and 46% less risk of death compared to patients receiving only CRT (Fig. 3a–c). We observed statistically significant qualitative interaction between treatment and HIF1α status for OS, which was validated by bootstrap-resampling method. We did not observe any prognostic and/or predictive association of EGFR, pEGFR dimers or EGFR gene copy number. Ours is the first study demonstrating both prognostic and predictive roles of HIF1α in HPV-negative LA-HNSCC patients.

HNSCCs are characterised by EGFR overexpression that is the principal mechanism of receptor activation; however, protein expression or gene copy number of EGFR have not emerged as a strong predicting biomarker for anti-EGFR-based treatment response.^9^ In this study, we found high EGFR expression to be associated with improved outcomes with NCRT versus CRT; however, the treatment interaction test was nonsignificant. Lack of correlation between EGFR-based biomarkers and sensitivity of EGFR inhibitors can be due to complex biology of the EGFR signalling pathways in which different intrinsic and extrinsic or acquired resistance mechanisms can alter EGFR downstream signalling. Potential mechanisms proposed for anti-EGFR therapy resistance are overexpression of ligands, activation of alternative pathways like ERBB2 and MET and/or alterations in downstream pathways due to mutations in PI3KCA, PTEN, RAS and CCND1 gene amplification. Resistance mechanisms of anti-EGFR treatment are well established in CRC and NSCLC, but are poorly understood and are not well established in HNSCCs.^34,35^

Interestingly, several preclinical studies on different cancer cell lines have repeatedly demonstrated that the response of tumour cells to EGFR inhibitors is linked to the downregulation of HIF1α.^16–19,36,37^ In vivo studies have further shown that this downregulation of HIF1α upon treatment with EGFR inhibitors decreases the levels of its downstream target vascular endothelial growth factor (VEGF), a strong pre-angiogenic marker, which in turn causes vascular normalisation and improved blood flow leading to enhanced chemoradiation efficacy.^17,19^ Nevertheless, the predictive impact of HIF1α or VEGF in response to anti-EGFR-based treatments in HNSCC patients has not been studied. A retrospective study by Ou et al. has reported an independent prognostic role of combined expression of low CD34 and high CA9 in predicting poor LCR; however, no predictive effect of these hypoxia-based biomarkers was observed in HPV-negative LA-HNSCC patients. This study was, however, carried out in a small number of patients with an unbalanced distribution of patients between the two treatment groups.^38^ Ours is the first study demonstrating the role of high nuclear HIF1α expression in predicting poor response to cisplatin radiation and significant better treatment response in high HIF1α-expressing patients upon addition of nimotuzumab to cisplatin radiation. In addition, a study by Boeckx et al. showed increased sensitivity of HNSCC cells to cetuximab under hypoxia.^21^ Similar observations were also reported by Wiechec et al., they further showed that the sensitivity to cetuximab was efficiently reversed by knockdown of HIF1α in HNSCC cells.^22^ However, the underlying mechanism by which hypoxia or HIF1α mediates sensitisation towards anti-EGFR mAbs is not yet clearly understood. Our combined analysis of EGFR and HIF1α revealed that improved treatment response to NCRT was independent of EGFR expression status.

In this study, we have used RNA-ISH as a confirmatory test for detecting transcriptionally active HPV, unlike the majority of the biomarker studies in which HPV detection is solely done by p16 IHC that is a surrogate marker and not specific for detecting biologically active HPV.^24^ HNSCC tumours with HPV-positive status are genetically and biologically distinct from HPV-negative tumours,^39–42^ and are associated with better outcomes, irrespective of the treatment modalities.^43–45^ To maintain the homogeneity of our study group, we excluded these HPV-positive cases from the current analysis. Also, due to low HPV prevalence in our cohort, we could not perform an independent prognostic and predictive biomarker analysis in the HPV-positive subgroup. There are however few limitations of this study, which need to be considered. IHC staining was assessed semi-quantitatively; evaluation of membrane-staining intensity and quantification is inherently subjective. In addition, ours is a single-centre study, and therefore the results need multicentric external validation.

Since hypoxia is a dynamic feature of tumour microenvironment, assessing biomarker expression in biopsy specimens might not be representative of the whole tumour. In addition, integrating functional imaging and serum-based biomarker analysis can offer complementary information on development of robust predictive biomarkers. However, very few reports have studied correlations between tissues or serum-based biomarkers and information obtained from functional imaging. Recently, Nicolay et al. in a prospective study have shown the association of tumour hypoxia markers—HIF1α and CA9—studied by IHC in pre-treatment biopsies with the hypoxia dynamics during chemoradiation assessed by 18F-FMISO PET/CT imaging in LA-HNSCC patients.^46^ In addition to hypoxia and angiogenic markers, other frequently altered downstream molecules of EGFR signalling, including the PI3K–AKT–mTOR pathway, need to be evaluated in combination for their predictive potential in HNSCCs.^47,48^

In conclusion, our results suggest that high nuclear HIF1α expression is associated with poor clinical outcomes in CRT-treated patients. Addition of nimotuzumab to CRT significantly improves the outcomes in high HIF1α-expressing patients. In addition to nimotuzumab, anti-angiogenic drugs can be explored for high HIF1α-expressing patients.^49^ These targeted therapies are frequently associated with different levels of toxicity and often expensive; therefore, it is required to identify the patients upfront who are most likely to be benefited from these treatments.

## Supplementary information

Supplementary information

## Data Availability

All data generated or analysed during this study are included in this published article (and its supplementary information file). However, if required, we can submit the clinical outcomes/follow-up and biomarker data.

## References

[1] Bray F, Ferlay J, Soerjomataram I, Siegel RL, Torre LA, Jemal A (2018). Global cancer statistics 2018: GLOBOCAN estimates of incidence and mortality worldwide for 36 cancers in 185 countries. CA Cancer J. Clin..

[2] Leemans CR, Braakhuis BJ, Brakenhoff RH (2011). The molecular biology of head and neck cancer. Nat. Rev. Cancer.

[3] Tian Y, Lin J, Tian Y, Zhang G, Zeng X, Zheng R (2018). Efficacy and safety of anti-EGFR agents administered concurrently with standard therapies for patients with head and neck squamous cell carcinoma: a systematic review and meta-analysis of randomized controlled trials. Int. J. Cancer.

[4] Allan DG (2005). Nimotuzumab: evidence of clinical benefit without rash. Oncologist..

[5] Ramakrishnan MS, Eswaraiah A, Crombet T, Piedra P, Saurez G, Iyer H (2009). Nimotuzumab, a promising therapeutic monoclonal for treatment of tumors of epithelial origin. mAbs.

[6] Patil VM, Noronha V, Joshi A, Agarwal J, Ghosh-Laskar S, Budrukkar A (2019). A randomized phase 3 trial comparing nimotuzumab plus cisplatin chemoradiotherapy versus cisplatin chemoradiotherapy alone in locally advanced head and neck cancer. Cancer.

[7] Amado RG, Wolf M, Peeters M, Van Cutsem E, Siena S, Freeman DJ (2008). Wild-type KRAS is required for panitumumab efficacy in patients with metastatic colorectal cancer. J. Clin. Oncol..

[8] Rosell R, Moran T, Queralt C, Porta R, Cardenal F, Camps C (2009). Screening for epidermal growth factor receptor mutations in lung cancer. N. Engl. J. Med..

[9] Bossi P, Resteghini C, Paielli N, Licitra L, Pilotti S, Perrone F (2016). Prognostic and predictive value of EGFR in head and neck squamous cell carcinoma. Oncotarget.

[10] Batzer AG, Rotin D, Urena JM, Skolnik EY, Schlessinger J (1994). Hierarchy of binding sites for Grb2 and Shc on the epidermal growth factor receptor. Mol. Cell Biol..

[11] Harris AL (2002). Hypoxia–a key regulatory factor in tumour growth. Nat. Rev. Cancer.

[12] Brown JM (1999). The hypoxic cell: a target for selective cancer therapy–eighteenth Bruce F. Cain Memorial Award lecture. Cancer Res..

[13] Vaupel P, Mayer A (2007). Hypoxia in cancer: significance and impact on clinical outcome. Cancer Metastasis Rev..

[14] Semenza GL (2012). Hypoxia-inducible factors: mediators of cancer progression and targets for cancer therapy. Trends Pharmacol. Sci..

[15] Gong L, Zhang W, Zhou J, Lu J, Xiong H, Shi X (2013). Prognostic value of HIFs expression in head and neck cancer: a systematic review. PLoS ONE.

[16] Li X, Lu Y, Liang K, Pan T, Mendelsohn J, Fan Z (2008). Requirement of hypoxia-inducible factor-1alpha down-regulation in mediating the antitumor activity of the anti-epidermal growth factor receptor monoclonal antibody cetuximab. Mol. Cancer Ther..

[17] Cerniglia GJ, Pore N, Tsai JH, Schultz S, Mick R, Choe R (2009). Epidermal growth factor receptor inhibition modulates the microenvironment by vascular normalization to improve chemotherapy and radiotherapy efficacy. PLoS ONE.

[18] Li X, Fan Z (2010). The epidermal growth factor receptor antibody cetuximab induces autophagy in cancer cells by downregulating HIF-1alpha and Bcl-2 and activating the beclin 1/hVps34 complex. Cancer Res..

[19] Wang WM, Zhao ZL, Ma SR, Yu GT, Liu B, Zhang L (2015). Epidermal growth factor receptor inhibition reduces angiogenesis via hypoxia-inducible factor-1alpha and Notch1 in head neck squamous cell carcinoma. PLoS ONE.

[20] Luwor RB, Lu Y, Li X, Mendelsohn J, Fan Z (2005). The antiepidermal growth factor receptor monoclonal antibody cetuximab/C225 reduces hypoxia-inducible factor-1 alpha, leading to transcriptional inhibition of vascular endothelial growth factor expression. Oncogene.

[21] Boeckx C, Van den Bossche J, De Pauw I, Peeters M, Lardon F, Baay M (2015). The hypoxic tumor microenvironment and drug resistance against EGFR inhibitors: preclinical study in cetuximab-sensitive head and neck squamous cell carcinoma cell lines. BMC Res. Notes.

[22] Wiechec, E., Hansson, K. T., Alexandersson, L., Jonsson, J. I. & Roberg K. Hypoxia mediates differential response to anti-EGFR therapy in HNSCC cells. *Int. J. Mol. Sci*. **18**, 943 (2017). 10.3390/ijms18050943 PMC5454856 28468237

[23] Bhosale PG, Pandey M, Desai RS, Patil A, Kane S, Prabhash K (2016). Low prevalence of transcriptionally active human papilloma virus in Indian patients with HNSCC and leukoplakia. Oral Surg. Oral Med. Oral Pathol. Oral Radiol..

[24] Craig SG, Anderson LA, Schache AG, Moran M, Graham L, Currie K (2019). Recommendations for determining HPV status in patients with oropharyngeal cancers under TNM8 guidelines: a two-tier approach. Br. J. Cancer.

[25] Chung CH, Ely K, McGavran L, Varella-Garcia M, Parker J, Parker N (2006). Increased epidermal growth factor receptor gene copy number is associated with poor prognosis in head and neck squamous cell carcinomas. J. Clin. Oncol..

[26] Keren S, Shoude Z, Lu Z, Beibei Y (2014). Role of EGFR as a prognostic factor for survival in head and neck cancer: a meta-analysis. Tumour Biol..

[27] Swartz JE, Pothen AJ, Stegeman I, Willems SM, Grolman W (2015). Clinical implications of hypoxia biomarker expression in head and neck squamous cell carcinoma: a systematic review. Cancer Med..

[28] Clark GM (2008). Prognostic factors versus predictive factors: examples from a clinical trial of erlotinib. Mol. Oncol..

[29] Polley MY, Freidlin B, Korn EL, Conley BA, Abrams JS, McShane LM (2013). Statistical and practical considerations for clinical evaluation of predictive biomarkers. J. Natl Cancer Inst..

[30] Bland JM, Altman DG (1986). Statistical methods for assessing agreement between two methods of clinical measurement. Lancet.

[31] Lin LI (1989). A concordance correlation coefficient to evaluate reproducibility. Biometrics.

[32] McShane LM, Altman DG, Sauerbrei W, Taube SE, Gion M, Clark GM (2005). REporting recommendations for tumour MARKer prognostic studies (REMARK). Br. J. Cancer.

[33] Altman DG, McShane LM, Sauerbrei W, Taube SE (2012). Reporting recommendations for tumor marker prognostic studies (REMARK): explanation and elaboration. PLoS Med..

[34] Boeckx C, Baay M, Wouters A, Specenier P, Vermorken JB, Peeters M (2013). Anti-epidermal growth factor receptor therapy in head and neck squamous cell carcinoma: focus on potential molecular mechanisms of drug resistance. Oncologist.

[35] Chen LF, Cohen EE, Grandis JR (2010). New strategies in head and neck cancer: understanding resistance to epidermal growth factor receptor inhibitors. Clin. Cancer Res..

[36] Lu Y, Liang K, Li X, Fan Z (2007). Responses of cancer cells with wild-type or tyrosine kinase domain-mutated epidermal growth factor receptor (EGFR) to EGFR-targeted therapy are linked to downregulation of hypoxia-inducible factor-1alpha. Mol. Cancer.

[37] Pore N, Jiang Z, Gupta A, Cerniglia G, Kao GD, Maity A (2006). EGFR tyrosine kinase inhibitors decrease VEGF expression by both hypoxia-inducible factor (HIF)-1-independent and HIF-1-dependent mechanisms. Cancer Res..

[38] Ou D, Garberis I, Adam J, Blanchard P, Nguyen F, Levy A (2018). Prognostic value of tissue necrosis, hypoxia-related markers and correlation with HPV status in head and neck cancer patients treated with bio- or chemo-radiotherapy. Radiother. Oncol..

[39] Stransky N, Egloff AM, Tward AD, Kostic AD, Cibulskis K, Sivachenko A (2011). The mutational landscape of head and neck squamous cell carcinoma. Science.

[40] Smeets SJ, Braakhuis BJ, Abbas S, Snijders PJ, Ylstra B, van de Wiel MA (2006). Genome-wide DNA copy number alterations in head and neck squamous cell carcinomas with or without oncogene-expressing human papillomavirus. Oncogene.

[41] Slebos RJ, Yi Y, Ely K, Carter J, Evjen A, Zhang X (2006). Gene expression differences associated with human papillomavirus status in head and neck squamous cell carcinoma. Clin. Cancer Res..

[42] Dok, R. & Nuyts, S. HPV positive head and neck cancers: molecular pathogenesis and evolving treatment strategies. *Cancers* **8**, 41 (2016). 10.3390/cancers8040041 PMC4846850 27043631

[43] Ang KK, Harris J, Wheeler R, Weber R, Rosenthal DI, Nguyen-Tan PF (2010). Human papillomavirus and survival of patients with oropharyngeal cancer. N. Engl. J. Med..

[44] Tian S, Switchenko JM, Jhaveri J, Cassidy RJ, Ferris MJ, Press RH (2019). Survival outcomes by high-risk human papillomavirus status in nonoropharyngeal head and neck squamous cell carcinomas: a propensity-scored analysis of the national cancer data base. Cancer.

[45] Li H, Torabi SJ, Yarbrough WG, Mehra S, Osborn HA, Judson B (2018). Association of human papillomavirus status at head and neck carcinoma subsites with overall survival. JAMA Otolaryngol. Head Neck Surg..

[46] Nicolay NH, Wiedenmann N, Mix M, Weber WA, Werner M, Grosu AL (2020). Correlative analyses between tissue-based hypoxia biomarkers and hypoxia PET imaging in head and neck cancer patients during radiochemotherapy-results from a prospective trial. Eur. J. Nucl. Med. Mol. Imaging.

[47] Eze N, Lee JW, Yang DH, Zhu F, Neumeister V, Sandoval-Schaefer T (2019). PTEN loss is associated with resistance to cetuximab in patients with head and neck squamous cell carcinoma. Oral Oncol..

[48] Lui VW, Hedberg ML, Li H, Vangara BS, Pendleton K, Zeng Y (2013). Frequent mutation of the PI3K pathway in head and neck cancer defines predictive biomarkers. Cancer Discov..

[49] Micaily I, Johnson J, Argiris A (2020). An update on angiogenesis targeting in head and neck squamous cell carcinoma. Cancers Head Neck.

