# Supplementary material for: Prognostic and predictive significance of nuclear HIF1α expression in locally advanced HNSCC patients treated with chemoradiation with or without nimotuzumab
Source: Br J Cancer. 2020 Sep 17;123(12):1757–66. doi: 10.1038/s41416-020-01064-4 (PMC7722894; doi:10.1038/s41416-020-01064-4)
Supplement: Supplementary file 1 — Supplementary information [file 41416_2020_1064_MOESM1_ESM.pdf]

SUPPLEMENTARY INFORMATION

Prognostic and predictive significance of nuclear HIF1α expression in locally-advanced HNSCC patients treated with chemo-radiation with or without nimotuzumab

Patel et al.

SUPPLEMENTARY METHODS

Brief assay protocols

p16 immunohistochemistry (IHC)

p16 status was evaluated by IHC analysis using CINtec Histology Kit (Roche mtm laboratories AG, Heidelberg, Germany) according to the manufacturer's instructions. FFPE tissue sections of known p16 positive cervical cancer were used as positive control in every run. Detailed protocol is as previously published [1]. p16 IHC was scored positive if there was strong to moderate diffused nuclear and cytoplasmic staining present in >70% of the tumor cells. In addition to p16 positive samples, p16 negative samples showing >5% p16 positivity in tumor cells were also tested for HPV RNA-ISH.

Extraction of genomic DNA from paraffin-embedded tumor tissue and saliva

Genomic DNA was extracted from FFPE tissues by column purification using QIAamp DNA FFPE Tissue Kit (Qiagen, Hilden, Germany) according to the manufacturer's instructions. Genomic DNA from saliva was extracted according to the manufacturer's instructions (DNA Genotek Inc., Ontario, Canada). Briefly, saliva samples were heated at 56°C for 6 h. 500uL of saliva was treated with purifier (OG-L2P) to obtain clear supernatant which was subjected to absolute ethanol treatment for DNA precipitation, after washing step with 70% ethanol, DNA pellet was dissolved in autoclaved milliQ water. Detailed protocol is as previously published [1].

Nested PCR

Genomic DNA extracted from FFPE tissue and saliva was subjected PCR using MY09/MY11 primers. PCR product of this PCR (450bp) was amplified using GP5+/GP6+ primers by touch down PCR. Diluted DNA (0.05ng/µL) from HeLa cell line (Cervical cancer cell line, HPV18 positive) was used as positive control. Care was taken to avoid cross contamination between the samples and the positive control. PCR positive cases (either tissue or saliva) were tested for transcriptionally active HPV by RNA ISH. Detailed protocol is as previously published [1].

RNA *in-situ* hybridization (RNA ISH)

Detection of E6/E7 mRNA of seven high-risk (HR) HPV (HPV16, 18, 31, 33, 35, 52, and 58) was performed using the RNAscope ® 2.5 HD assay with Brown HPV HR7 Kit (Advanced Cell Diagnostics Inc. CA, USA) as per manufacturer's protocol. Probe against ubiquitin C (UBC) was used as internal positive control and prob against bacterial gene dapB was used as internal negative control. Detailed protocol is as previously published [1].

Detailed assay protocols

Fluorescence *in situ* hybridization (FISH)

Five µm FFPE tissue sections were baked at 40°C for 2 hours ,deparaffinized, dehydrated and treated with 2.5 % Sodium thiocyanate for 5 min. Followed by heat treatment in 10mM Sodium citrate buffer (pH 5.8) for 4 min in 700-W microwave oven (2,450 MHz; LG MS2043DB; LG Electronics, India). Followed by, digestion treatment with pepsin (0.2 gm in 50 ml of pre-warmed 10mM HCl) for 40 min at 37°C in moist chamber. After passing through ethanol series and drying, probes (EGFR SpectrumOrange and CEP7 SpectrumGreen) were added and codenaturation was performed at 80°C for 5 min and probes were allowed to hybridize at 37°C for 18-20 hrs in a moist chamber. After post-hybridization washes and drying, chromatin counterstaining was done using DAPI Vectashield mounting medium. FISH signals were counted using Metasystems software in the Zeiss Axioscope fluorescence microscope.

Immunohistochemistry (IHC)

Five µm FFPE tissue sections were baked at 60°C for 20 min followed by de-paraffinization in xylene and rehydration in graded alcohol. Heat-induced epitope retrieval (HIER) was performed using 700-W microwave oven at highest power in appropriate buffer (Supplementary table S1). Slides were allowed to cool down to room temperature (RT) followed by washing with PBS. Endogenous peroxidase activity was blocked by 3% hydrogen peroxide for 30 min at RT. After washing with PBS blocking was done using normal horse serum (Vector Laboratories, Burlingame, CA, USA) at RT for 60 min followed by overnight incubation with respective primary antibody at 4°C (Supplementary table S1). Next day, sections were washed with PBST (PBS with 0.1% Tween 20) and incubated with biotinylated universal secondary antibody (Vector Laboratories, Burlingame, CA, USA) for 40 min at RT followed by washing with PBST and incubation with avidin-biotin-peroxidase complex (Vector Laboratories, Burlingame, CA, USA) for 40 min at RT. After washing immuno staining was developed using DAB chromogen (diaminobenzidine, Sigma-Aldrich). Sections were then counterstained with hematoxylin, dehydrated in graded alcohols, cleared in xylene, and mounted in DPX. Each staining batch involved a respective positive control and negative control stained with rabbit IgG isotype control antibody (abcam, ab172730) at the same dilution as of primary antibody. Details of epitope retrieval buffer, serum blocking percentage, primary antibody and positive control used are provided in (Supplementary Table S1).

Reference:

1. Bhosale PG, Pandey M, Desai RS, et al. Low prevalence of transcriptionally active human papilloma virus in Indian patients with HNSCC and leukoplakia. *Oral Surg Oral Med Oral Pathol Oral Radiol* 2016; 122: 609-618 e607. 2016/10/22. DOI: 10.1016/j.oooo.2016.06.006.

SUPPLEMENTARY TABLES

Supplementary Table 1: Primary antibody, retrieval buffer and positive control used for immunohistochemistry

Biomarker	Primary antibody			Epitope retrieval buffer	Serum blocking	Positive control
	Source	Host species, clone	Dilution			
HIF1α	Novus Biologicals (NB100-479)	Rabbit polyclonal	1:300	1 mM EDTA, pH 8	5%	Human renal cell carcinoma FFPE tissue
EGFR	Cell Signaling Tec (#4267)	Rabbit, D38B1	1:50	10 mM Tris - 1mM EDTA, pH 9	1%	Human lung cancer FFPE tissue
pEGFRY1068	Cell Signaling Tec (#3777)	Rabbit, D7A5	1:100	1 mM EDTA, pH 8	2%	SignalSlide™ Cell Signaling Tec (Cat no- #8102)
pEGFRY1173	Novus Biologicals (NB110-56948)	Rabbit, E124	1:400	1 mM EDTA, pH 8	2%	SignalSlide™ Cell Signaling Tec (Cat no- #8102)

FFPE= Formalin fixed paraffin embedded; EDTA=Ethylene diamine tetra acetic acid

Supplementary Table 2: EGFR-FISH signal categorization and distribution of the patients

Categories	Definition	CRT (n=143)	NCRT (n=148)
Disomy	≤2 copies in >90% cells	6 (4.2)	6 (4.0)
Trisomy	3 copies in ≥10% cells or ≥4 copies in <10% cells	5 (3.5)	5 (3.4)
Low polysomy	≥4 copies in 10% -40% cells	84 (58.7)	90 (60.8)
High polysomy	≥4 copies in ≥40% cells	14 (9.8)	17 (11.5)
Amplification	ratio of the EGFR gene to chromosome 7 of ≥2, or ≥15 copies of EGFR per cell in ≥10% of cells	34 (23.8)	30 (20.3)

*Pearson's $\chi 2$ Square test; Data are number (%); Disomy, trisomy and low polysomy are categorized as EGFR FISH negative; high polysomy and amplification are categorized as EGFR FISH positive. NCRT=nimotuzumab plus cisplatin-radiation; CRT=cisplatin-radiation alone.

Supplementary Table 3: Correlation among different biomarkers

		EGFR (Membrane)		EGFR (Cytoplasm)		pEGFRY1068		pEGFRY1173		EGFR-FISH	
		**Low**	**High**	**Low**	**High**	**Negative**	**Positive**	**Negative**	**Positive**	**Negative**	**Positive**
**HIF1α**	**Low, n (%)**	115 (57.5)	85 (42.5)	108 (54)	92 (46)	167 (85.2)	29 (14.8)	147 (77.8)	42 (22.2)	87 (62.1)	53 (37.9)
	**High, n (%)**	82 (42.7)	110 (57.3)	113 (58.9)	79 (41.1)	152 (81.3)	35 (18.7)	127 (70.2)	54 (29.8)	107 (71.8)	42 (28.2)
	P*	0.003		0.333		0.304		0.095		0.080	
	**R**	0.15		-0.049		0.053		0.087		-0.10	
**EGFR (Membrane)**	**Low, n (%)**			156 (76.5)	48 (23.5)	182 (92.9)	14 (7.1)	164 (86.3)	26 (13.7)	110 (73.8)	39 (26.2)
	**High, n (%)**			73 (36.5)	127 (63.5)	146 (74.1)	51 (25.9)	116 (62.4)	70 (37.6)	86 (60.6)	56 (39.4)
	P*			0.000		0.000		0.000		0.016	
	**R**			0.40		0.25		0.28		0.14	
**EGFR (Cytoplasm)**	**Low, n (%)**					198 (89.6)	23 (10.4)	167 (79.9)	42 (20.1)	119 (72.6)	45 (27.4)
	**High, n (%)**					130 (75.6)	42 (24.4)	113 (67.7)	54 (32.3)	77 (60.6)	50 (39.4)
	P*					0.000		0.007		0.031	
	**R**					0.19		0.14		0.13	
**pEGFRY1068**	**Negative, n (%)**							269 (85.4)	46 (14.6)	178 (73.6)	64 (26.4)
	**Positive, n (%)**							11 (18)	50 (82)	16 (35.6)	29 (64.4)
	P*							0.000		0.000	
	**R**							0.57		0.30	
**pEGFRY1173**	**Negative, n (%)**									158 (74.5)	54 (25.5)
	**Positive, n (%)**									29 (44.6)	36 (55.4)
	P*									0.000	
	**R**									0.27	

(*) Pearson's χ2 tests; R=Pearson correlation coefficient

Supplementary Table 4: Association between biomarkers and patient's clinical characteristics

		Age (Years)		Gender		Tobacco-alcohol habit		Site of tumor		Clinical stage	
		**Below 60**	**Above 60**	**Male**	**Female**	**No habit**	**With habit**	**Oropharynx**	**Others**	**III**	**IV**
**HIF1α**	**Low, n (%)**	136 (68.0)	64 (32.0)	171 (85.5)	29 (14.5)	10 (5.1)	185 (94.9)	94 (47.0)	106 (53.0)	43 (21.5)	157 (78.5)
	**High, n (%)**	130 (67.7)	62 (32.3)	172 (89.6)	20 (10.4)	17 (9.0)	171 (91.0)	93 (48.4)	99 (51.6)	52 (27.1)	140 (72.9)
	P*	0.951		0.222		0.135		0.776		0.197	
**EGFR (Membrane)**	**Low, n (%)**	139 (68.1)	65 (31.9)	184 (90.2)	20 (9.8)	14 (7.1)	184 (92.9)	99 (48.5)	105 (51.5)	47 (23.0)	157 (77.0)
	**High, n (%)**	138 (69.0)	62 (31.0)	168 (84.0)	32 (16.0)	16 (8.20)	179 (91.8)	97 (48.5)	103 (51.5)	51 (25.5)	149 (74.5)
	P*	0.852		0.063		0.672		0.995		0.564	
**EGFR (Cytoplasm)**	**Low, n (%)**	157 (68.6)	72 (31.4)	204 (89.1)	25 (10.9)	16 (7.2)	205 (92.8)	111 (48.5)	118 (51.5)	65 (28.4)	164 (71.6)
	**High, n (%)**	120 (68.6)	55 (31.4)	148 (84.6)	27 (15.4)	14 (8.1)	158 (91.9)	85 (48.6)	90 (51.4)	33 (18.9)	142 (81.1)
	P*	0.998		0.18		0.739		0.984		**0.027**	
**pEGFRY1068**	**Negative, n (%)**	228 (69.5)	100 (30.5)	285 (86.9)	43 (13.1)	27 (8.5)	291 (91.5)	162 (49.4)	166 (50.6)	78 (23.8)	250 (76.2)
	**Positive, n (%)**	42 (64.6)	23 (35.4)	56 (86.2)	9 (13.8)	3 (4.7)	61 (95.3)	30 (46.2)	35 (53.8)	17 (26.2)	48 (73.8)
	P*	0.437		0.873		0.302		0.633		0.683	
**pEGFRY1173**	**Negative, n (%)**	193 (68.9)	87 (31.1)	240 (85.7)	40 (14.3)	23 (8.5)	249 (91.5)	133 (47.5)	147 (52.5)	71 (25.4)	209 (74.6)
	**Positive, n (%)**	65 (67.7)	31 (32.3)	86 (89.6)	10 (10.4)	6 (6.3)	89 (93.7)	52 (54.2)	44 (45.8)	19 (19.8)	77 (80.2)
	P*	0.824		0.335		0.506		0.26		0.27	
**EGFR-FISH**	**Negative, n (%)**	137 (69.9)	59 (30.1)	167 (85.2)	29 (14.8)	19 (9.7)	176 (90.3)	99 (50.5)	97 (49.5)	47 (24.0)	149 (76.0)
	**Positive, n (%)**	63 (66.3)	32 (33.7)	83 (87.4)	12 (12.6)	4 (4.3)	90 (95.7)	41 (43.2)	54 (56.8)	22 (23.2)	73 (76.8)
	P*	0.537		0.619		0.43		0.239		0.877	

(*) Pearson's χ2 tests. Clinical stage is according to AJCC-UICC system (8th edition).

Supplementary Table 5 (A): Cutpoint analysis to assess prognostic effect of HIF1α H-score

CRT (n=199)			PFS		LRC		OS	
Cut point	Low (n)	High (n)	HR (95% CI)	*P**	HR (95% CI)	*P**	HR (95% CI)	*P**
≤10 &>10	28	171	0.75 (0.41-1.37)	0.348	0.48 (0.22-1.03)	0.06	0.51 (0.25-1.05)	0.069
≤30 &>30	47	152	0.50 (0.28-0.88)	0.015	0.52 (0.29-0.94)	0.029	0.50 (0.28-0.88)	0.015
≤50 &>50	71	128	0.69 (0.45-1.05)	0.086	0.53 (0.33-0.87)	0.011	0.74 (0.48-1.13)	0.164
≤70 &>70	82	117	0.74 (0.50-1.11)	0.143	0.63 (0.40-0.99)	0.045	0.80 (0.54-1.20)	0.281
**≤90 &>90**	**108**	**91**	**0.69 (0.47-1.01)**	**0.053**	**0.58 (0.38-0.89)**	**0.011**	**0.62 (0.42-0.91)**	**0.016**
≤120 &>120	120	79	0.79 (0.54-1.16)	0.231	0.72 (0.47-1.10)	0.13	0.72 (0.49-1.05)	0.089
≤160 &>160	138	61	0.74 (0.50-1.12)	0.153	0.67 (0.43-1.04)	0.073	0.62 (0.42-0.93)	0.02
≤180 &>180	155	44	0.74 (0.47-1.17)	0.197	0.64 (0.40-1.04)	0.07	0.66 (0.43-1.02)	0.062
≤240 &>240	169	30	0.59 (0.36-0.96)	0.035	0.48 (0.29-0.82)	0.006	0.51 (0.32-0.82)	0.005

*Univariate Cox regression analysis. Results at median cut point are highlighted in bold. CRT=cisplatin-radiation alone; HR=hazard ratio; CI=confidence interval; PFS=progression free survival; LRC=loco-regional control; OS=overall survival.

Supplementary Table 5 (B): Cutpoint analysis to assess prognostic effect of membrane EGFR H-score

CRT (n=206)			PFS		LRC		OS	
**Cut point**	**Low (n)**	**High (n)**	**HR (95% CI)**	***P****	**HR (95% CI)**	***P****	**HR (95% CI)**	***P****
≤10 & >10	35	171	1.18 (0.73-1.92)	0.503	1.19 (0.70-2.01)	0.529	0.98 (0.59-1.63)	0.947
≤20 & >20	46	160	0.95 (0.60-1.49)	0.817	1.01 (0.62-1.64)	0.983	0.84 (0.52-1.35)	0.466
≤40 & >40	59	147	0.93 (0.61-1.41)	0.727	0.93 (0.59-1.47)	0.758	0.91 (0.60-1.38)	0.65
≤60 & >60	73	133	0.87 (0.59-1.30)	0.499	0.87 (0.56-1.35)	0.541	0.86 (0.57-1.28)	0.445
≤80 & >80	85	121	0.87 (0.59-1.28)	0.488	0.84 (0.55-1.28)	0.411	0.92 (0.62-1.35)	0.667
**≤100 & >100**	**103**	**103**	**0.84 (0.58-1.23)**	**0.37**	**0.79 (0.52-1.20)**	**0.264**	**0.83 (0.57-1.22)**	**0.348**
≤120 & >120	119	87	0.90 (0.61-1.32)	0.584	0.92 (0.60-1.40)	0.687	0.96 (0.65-1.41)	0.816
≤140 & >140	141	65	0.87 (0.58-1.30)	0.492	0.99 (0.63-1.56)	0.973	0.90 (0.60-1.36)	0.621
≤160 & >160	158	48	0.90 (0.57-1.41)	0.642	1.12 (0.67-1.88)	0.67	1.02 (0.64-1.63)	0.927
≤200 & >200	177	29	0.89 (0.52-1.51)	0.656	1.16 (0.62-2.19)	0.644	1.04 (0.60-1.79)	0.90
≤240 & >240	186	20	0.61 (0.34-1.09)	0.094	0.82 (0.41-1.64)	0.576	0.83 (0.44-1.55)	0.557

*Univariate Cox regression analysis. Results at median cut point are highlighted in bold. CRT=cisplatin-radiation alone; HR=hazard ratio; CI=confidence interval; PFS=progression free survival; LRC=loco-regional control; OS=overall survival.

Supplementary Table 5 (C) :Cutpoint analysis to assess prognostic effect of cytoplasmic EGFR H-score

CRT (n=206)			PFS		LRC		OS	
**Cut point**	**Low (n)**	**High (n)**	**HR (95% CI)**	***P****	**HR (95% CI)**	***P****	**HR (95% CI)**	***P****
≤20 & >20	15	191	1.07 (0.54-2.12)	0.845	1.35 (0.68-2.70)	0.389	0.80 (0.37-1.72)	0.561
≤40 & >40	30	176	1.01 (0.60-1.72)	0.966	1.31 (0.76-2.24)	0.335	1.02 (0.60-1.73)	0.957
≤60 & >60	47	159	0.90 (0.57-1.42)	0.657	1.06 (0.66-1.72)	0.808	0.82 (0.51-1.32)	0.419
≤80 & >80	74	132	1.02 (0.69-1.50)	0.938	1.19 (0.78-1.81)	0.428	0.95 (0.64-1.41)	0.797
≤100 & >100	83	123	0.99 (0.67-1.45)	0.95	1.02 (0.67-1.55)	0.931	0.97 (0.66-1.43)	0.873
≤120 & >120	94	112	0.92 (0.63-1.35)	0.674	0.99 (0.66-1.50)	0.974	0.96 (0.65-1.40)	0.816
**≤140 & >140**	**121**	**85**	**0.82 (0.56-1.20)**	**0.299**	**0.90 (0.59-1.37)**	**0.626**	**1.03 (0.69-1.52)**	**0.901**
≤160 & >160	155	51	0.61 (0.40-0.91)	0.017	0.68 (0.43-1.08)	0.101	0.81 (0.53-1.26)	0.352
≤180 & >180	190	16	1.13 (0.52-2.43)	0.759	1.33 (0.54-3.27)	0.541	1.38 (0.61-3.14)	0.445
≤190 & >190	198	8	1.31 (0.41-4.13)	0.647	1.68 (0.41-6.82)	0.47	2.13 (0.53-8.64)	0.289

*Univariate Cox regression analysis. Results at median cut point are highlighted in bold. CRT=cisplatin-radiation alone; HR=hazard ratio; CI=confidence interval; PFS=progression free survival; LRC=loco-regional control; OS=overall survival

Supplementary Table 6: Univariate analysis of associations between biomarkers and clinical outcomes in CRT group

Variables		Progression free survival			Loco-regional control			Overall survival		
		**Events/n**	**HR (95% CI)**	***P****	**Events/n**	**HR (95% CI)**	***P****	**Events/n**	**HR (95% CI)**	***P****
**pEGFRY1068**	**Negative**	83/168	0.63 (0.40-0.99)	0.048	73/168	0.88 (0.51-1.54)	0.655	89/168	1.08 (0.64-1.84)	0.769
	**Positive**	23/32			15/32			16/32		
**pEGFRY1173**	**Negative**	69/142	0.74 (0.48-1.14)	0.17	61/142	0.93 (0.57-1.52)	0.766	74/142	1.06 (0.67-1.70)	0.801
	**Positive**	29/46			21/46			23/46		
**EGFR FISH**	**Negative**	51/95	1.05 (0.66-1.69)	0.827	40/95	0.94 (0.56-1.59)	0.829	56/95	1.11 (0.70-1.76)	0.651
	**Positive**	26/48			22/48			27/48		

*Univariate Cox regression analysis. n=number of patients; CRT=cisplatin-radiation alone; HR=hazard ratio; CI=confidence interval

Supplementary Table 7: Univariate analysis of associations between clinicopathological characteristics and clinical outcomes in CRT group

Characteristics		Progression free survival			Loco-regional control			Overall survival		
		Events/n	HR (95% CI)	*P**	Events/n	HR (95% CI)	*P**	Events/n	HR (95% CI)	*P**
**Age**	**Below 60**	82/148	1.46 (0.94-2.28)	0.092	69/148	1.49 (0.91-2.43)	0.111	83/148	1.59 (1.0-2.53)	0.049
	**Above 60**	26/58			21/58			23/58		
**Gender**	**Male**	95/181	0.96 (0.54-1.71)	0.884	79/181	0.95 (0.50-1.78)	0.867	93/181	1.0 (0.56-1.79)	1.00
	**Female**	13/25			11/25			13/25		
**Disease stage#**	**III**	20/58	0.48 (0.30-0.78)	0.003	15/58	0.43 (0.25-0.75)	0.003	24/58	0.64 (0.40-1.00)	0.051
	**IV**	88/148			75/148			82/148		
**Tumor site**	**Oropharynx**	62/96	1.74 (1.19-2.56)	0.004	50/96	1.58 (1.05-2.40)	0.03	58/96	1.62 (1.10-2.37)	0.014
	**Others**	46/110			40/110			48/110		

*Univariate Cox regression analysis. n=number of patients; CRT=cisplatin-radiation alone; HR=hazard ratio; CI=confidence interval; #According to AJCC-UICC system (8th edition).

Supplementary Table 8: Univariate analysis of associations between biomarkers and clinical outcomes in NCRT group

Variables		Progression free survival (PFS)			Loco-regional control (LRC)			Overall survival (OS)		
		Events/n	HR (95% CI)	*P**	Events/n	HR (95% CI)	*P**	Events/n	HR (95% CI)	*P**
**Age**	**Below 60**	54/129	1.12 (0.70-1.79)	0.64	45/129	1.25 (0.74-2.12)	0.402	60/129	1.10 (0.70-1.71)	0.688
	**Above 60**	26/69			20/69			29/69		
**Gender**	**Male**	71/171	1.31 (0.65-2.61)	0.45	58/171	1.38 (0.63-3.03)	0.42	78/171	1.12 (0.60-2.11)	0.717
	**Female**	9/27			7/27			11/27		
**Disease stage#**	**III**	8/40	0.33 (0.16-0.69)	**0.003**	8/40	0.42 (0.20-0.88)	**0.021**	10/40	0.40 (0.21-0.78)	**0.007**
	**IV**	72/158			57/158			79/158		
**Tumor site**	**Oropharynx**	46/100	1.49 (0.96-2.33)	0.079	41/100	1.97 (1.19-3.26)	**0.009**	51/100	1.55 (1.02-2.37)	**0.04**
	**Others**	34/98			24/98			38/98		
**HIF1α**	**Low**	38/92	1.08 (0.69-1.67)	0.747	28/92	0.87 (0.53-1.42)	0.577	47/92	1.36 (0.89-2.06)	0.153
	**High**	42/101			37/101			42/101		
**EGFR (M)**	**Low**	42/101	1.05 (0.68-1.63)	0.826	33/101	0.99 (0.61-1.62)	0.982	47/101	1.07 (0.71-1.62)	0.753
	**High**	38/97			32/97			43/97		
**EGFR (C)**	**Low**	45/108	1.06 (0.68-1.65)	0.801	40/108	1.36 (0.83-2.24)	0.227	48/108	1.04 (0.69-1.58)	0.858
	**High**	35/90			25/90			41/90		
**pEGFRY1068**	**Negative**	62/160	0.70 (0.4-1.23)	0.211	52/160	0.80 (0.42-1.54)	0.503	71/160	0.86 (0.48-1.52)	0.599
	**Positive**	15/33			Nov-33			14/33		
**pEGFRY1173**	**Negative**	58/138	0.98 (0.58-1.66)	0.93	48/138	1.02 (0.56-1.85)	0.945	64/138	1.00 (0.60-1.67)	0.991
	**Positive**	18/50			14/50			19/50		
**EGFR FISH**	**Negative**	41/101	0.80 (0.47-1.35)	0.394	36/101	1.02 (0.56-1.86)	0.953	49/101	0.82 (0.51-1.33)	0.431
	**Positive**	21/47			15/47			25/47		

*Univariate Cox regression analysis. n=number of patients; NCRT= nimotuzumab plus cisplatin-radiation; HR=hazard ratio; CI=confidence interval; #According to AJCC-UICC system (8th edition).

Supplementary Table 9: Cut point analysis to assess predictive effect of HIF1α H-score

**Low HIF1α H-score**						**High HIF1α H-score**						
**Progression free survival (PFS)**												
**Cut point**	**NCRT (n)**	**CRT (n)**	**4 year PFS (months)**	**HR (95% CI) (NCRT vs CRT)**	***P****	**Cut point**	**NCRT (n)**	**CRT (n)**	**4 year PFS (months)**	**HR (95% CI) (NCRT vs CRT)**	***P****	***P* (Interaction)**
≤10	23	28	45.8 vs 53.9	1.04 (0.44-2.47)	0.92	>10	170	171	52.8 vs 39.7	0.64 (0.47-0.87)	0.005	0.311
≤30	36	47	57.2 vs 56.9	0.82 (0.40-1.69)	0.588	>30	157	152	51.0 vs 37.2	0.64 (0.47-0.88)	0.006	0.579
≤50	51	71	56.7 vs 53.9	0.80 (0.45-1.44)	0.454	>50	142	128	50.6 vs 35.6	0.62 (0.44-0.87)	0.005	0.498
≤70	73	82	54.4 vs 49.8	0.76 (0.46-1.25)	0.277	>70	120	117	50.5 vs 36.3	0.63 (0.44-0.91)	0.012	0.558
**≤90**	**92**	**108**	**48.6 vs 46.4**	**0.84 (0.55-1.28)**	**0.422**	**>90**	**101**	**91**	**54.4 vs 35.6**	**0.55 (0.37-0.82)**	**0.003**	**0.137**
≤120	101	120	47.9 vs 43.1	0.79 (0.53-1.17)	0.235	>120	92	79	55.4 vs 39.0	0.57 (0.37-0.88)	0.011	0.253
≤160	128	138	48.4 vs 44.1	0.79 (0.55-1.12)	0.182	>160	65	61	58.0 vs 35.0	0.51 (0.31-0.85)	0.009	0.165
≤180	155	155	48.9 vs 42.7	0.76 (0.55-1.05)	0.10	>180	38	44	62.6 vs 36.8	0.47 (0.25-0.91)	0.026	0.149
≤240	166	169	49.0 vs 43.9	0.79 (0.58-1.08)	0.141	>240	27	30	69.3 vs 25.8	0.28 (0.12-0.65)	0.003	0.016
**Loco-regional control (LRC)**												
**Cut point**	**NCRT (n)**	**CRT (n)**	**4 year LRC (months)**	**HR (95% CI) (NCRT vs CRT)**	***P****	**Cut point**	**NCRT (n)**	**CRT (n)**	**4 year LRC (months)**	**HR (95% CI) (NCRT vs CRT)**	***P****	***P* (Interaction)**
≤10	23	28	57.1 vs 69.9	1.04 (0.33-3.29)	0.942	>10	170	171	58.9 vs 46.1	0.63 (0.45-0.88)	0.007	0.451
≤30	36	47	67.9 vs 68.0	0.70 (0.28-1.76)	0.45	>30	157	152	57.0 vs 43.9	0.64 (0.45-0.90)	0.01	0.877
≤50	51	71	67.5 vs 64.8	0.68 (0.33-1.41)	0.298	>50	142	128	56.1 vs 41.6	0.62 (0.43-0.89)	0.009	0.831
≤70	73	82	61.5 vs 58.7	0.73 (0.41-1.30)	0.289	>70	120	117	57.1 vs 43.0	0.62 (0.42-0.92)	0.017	0.613
**≤90**	**92**	**108**	**59.7 vs 56.0**	**0.80 (0.49-1.30)**	**0.363**	**>90**	**101**	**91**	**57.8 vs 41.0**	**0.55 (0.36-0.85)**	**0.006**	**0.234**
≤120	101	120	59.7 vs 51.5	0.70 (0.44-1.10)	0.122	>120	92	79	57.6 vs 45.5	0.61 (0.39-0.96)	0.034	0.633
≤160	128	138	57.6 vs 52.6	0.74 (0.50-1.11)	0.15	>160	65	61	60.2 vs 40.8	0.54 (0.31-0.92)	0.022	0.315
≤180	155	155	56.8 vs 51.4	0.75 (0.52-1.08)	0.126	>180	38	44	64.8 vs 41.1	0.48 (0.24-0.95)	0.035	0.181
≤240	166	169	56.8 vs 52.5	0.78 (0.55-1.11)	0.168	>240	27	30	69.3 vs 28.6	0.31 (0.13-0.72)	0.006	0.024

Supplementary Table 9: Cut point analysis to assess predictive effect of HIF1α H-score (continued)

Overall survival (OS)

Low HIF1α H-score						High HIF1α H-score						
**Cut point**	**NCRT (n)**	**CRT (n)**	**4 year OS (months)**	**HR (95% CI) (NCRT vs CRT)**	***P****	**Cut point**	**NCRT (n)**	**CRT (n)**	**4 year OS (months)**	**HR (95% CI) (NCRT vs CRT)**	***P****	***P* (Interaction)**
≤10	23	28	34.2 vs 64.4	2.26 (0.95-5.40)	0.066	>10	170	171	48.5 vs 38.4	0.68 (0.50-0.92)	0.011	0.009
≤30	36	47	39.2 vs 61.7	1.75 (0.88-3.50)	0.111	>30	157	152	48.4 vs 36.5	0.65 (0.47-0.89)	0.007	0.01
≤50	51	71	39.6 vs 48.6	1.18 (0.70-1.97)	0.532	>50	142	128	49.5 vs 37.7	0.65 (0.47-0.92)	0.014	0.053
≤70	73	82	42.7 vs 44.8	1.01 (0.64-1.59)	0.978	>70	120	117	49.4 vs 39.0	0.66 (0.46-0.95)	0.026	0.137
**≤90**	**92**	**108**	**41.2 vs 50.2**	**1.14 (0.76-1.70)**	**0.534**	**>90**	**101**	**91**	**52.1 vs 31.7**	**0.54 (0.36-0.81)**	**0.003**	**0.008**
≤120	101	120	40.0 vs 46.4	1.05 (0.72-1.53)	0.801	>120	92	79	53.9 vs 34.3	0.55 (0.36-0.84)	0.006	0.02
≤160	128	138	44.8 vs 45.9	0.99 (0.70-1.39)	0.93	>160	65	61	51.1 vs 31.4	0.50 (0.30-0.80)	0.004	0.017
≤180	155	155	45.1 vs 43.4	0.90 (0.65-1.23)	0.5	>180	38	44	54.1 vs 33.3	0.49 (0.26-0.90)	0.022	0.069
≤240	166	169	45.7 vs 44.7	0.90 (0.66-1.23)	0.517	>240	27	30	54.6 vs 23.1	0.37 (0.18-0.76)	0.007	0.017

*Univariate Cox regression analysis. Results at median cut point are highlighted in bold. CRT=cisplatin-radiation alone; NCRT=nimotuzumab plus cisplatin-radiation; HR=hazard ratio; CI=confidence interval.

Supplementary Table 10 (A): Cut point analysis to assess predictive effect of membrane EGFR H-score

Low EGFR H-score						High EGFR H-score						
**Progression free survival (PFS)**												
**Cut point**	**NCRT (n)**	**CRT (n)**	**4 year PFS (months)**	**HR (95% CI) (NCRT vs CRT)**	***P****	**Cut point**	**NCRT (n)**	**CRT (n)**	**4 year PFS (months)**	**HR (95% CI) (NCRT vs CRT)**	***P****	***P* (Interaction)**
≤10	29	35	63.2 vs 35.6	0.45 (0.21-0.95)	0.037	>10	169	171	50.6 vs 43.3	0.73 (0.53-1.0)	0.047	0.256
≤40	53	59	62.2 vs 42.4	0.54 (0.30-0.97)	0.037	>40	145	147	48.6 vs 42.1	0.73 (0.52-1.02)	0.064	0.408
≤60	74	73	59.4 vs 44.9	0.62 (0.37-1.01)	0.055	>60	124	133	48.4 vs 40.6	0.72 (0.50-1.02)	0.065	0.654
≤80	92	85	54.0 vs 43.8	0.73 (0.47-1.14)	0.165	>80	106	121	51.8 vs 41.0	0.64 (0.43-0.94)	0.022	0.591
**≤100**	**101**	**103**	**53.3 vs 44.2**	**0.75 (0.50-1.12)**	**0.156**	**>100**	**97**	**103**	**52.7 vs 40.0**	**0.61 (0.41-0.92)**	**0.02**	**0.46**
≤120	120	119	52.2 vs 42.2	0.72 (0.50-1.05)	0.085	>120	78	87	54.8 vs 42.0	0.61 (0.39-0.97)	0.038	0.537
≤140	133	141	50.7 vs 42.9	0.74 (0.52-1.05)	0.094	>140	65	65	58.2 vs 40.2	0.55 (0.33-0.93)	0.025	0.294
≤160	156	158	51.7 vs 42.8	0.71 (0.52-0.99)	0.041	>160	42	48	56.5 vs 38.4	0.56 (0.29-1.06)	0.072	0.435
≤180	166	168	51.5 vs 42.6	0.68 (0.50-0.94)	0.019	>180	32	38	57.0 vs 38.9	0.65 (0.32-1.32)	0.236	0.804
≤200	171	177	51.1 vs 43.2	0.69 (0.50-0.94)	0.018	>200	27	29	60.8 vs 36.6	0.63 (0.29-1.37)	0.245	0.786
≤240	175	186	51.1 vs 44.5	0.72 (0.53-0.97)	0.034	>240	23	20	62.4 vs 22.3	0.43 (0.18-1.01)	0.053	0.204
**Loco-regional control (LRC)**												
**Cut point**	**NCRT (n)**	**CRT (n)**	**4 year LRC (months)**	**HR (95% CI) (NCRT vs CRT)**	***P****	**Cut point**	**NCRT (n)**	**CRT (n)**	**4 year LRC (months)**	**HR (95% CI) (NCRT vs CRT)**	***P****	***P* (Interaction)**
≤10	29	35	71.1 vs 43.9	0.37 (0.15-0.89)	0.026	>10	169	171	57.2 vs 50.5	0.73 (0.52-1.03)	0.070	0.16
≤40	53	59	73.3 vs 51.3	0.43 (0.22-0.86)	0.016	>40	145	147	53.9 vs 48.8	0.75 (0.52-1.08)	0.120	0.17
≤60	74	73	69.5 vs 53.1	0.55 (0.32-0.97)	0.039	>60	124	133	52.9 vs 47.5	0.73 (0.49-1.07)	0.108	0.43
≤80	92	85	64.0 vs 53.6	0.70 (0.43-1.15)	0.16	>80	106	121	55.6 vs 46.6	0.63 (0.42-0.97)	0.034	0.75
**≤100**	**101**	**103**	**62.6 vs 54.0**	**0.74 (0.47-1.17)**	**0.193**	**>100**	**97**	**103**	**57.0 vs 45.1**	**0.59 (0.38-0.92)**	**0.021**	**0.48**
≤120	120	119	60.9 vs 50.4	0.69 (0.46-1.04)	0.078	>120	78	87	59.1 vs 48.4	0.62 (0.37-1.02)	0.060	0.70
≤140	133	141	58.8 vs 49.9	0.71 (0.48-1.03)	0.073	>140	65	65	63.7 vs 48.8	0.56 (0.31-1.01)	0.054	0.49
≤160	156	158	59.8 vs 49.7	0.65 (0.46-0.93)	0.019	>160	42	48	59.5 vs 48.2	0.69 (0.34-1.41)	0.306	0.93
≤180	166	168	59.0 vs 49.5	0.63 (0.45-0.90)	0.01	>180	32	38	60.9 vs 49.7	0.84 (0.38-1.87)	0.666	0.57

≤200	171	177	58.6 vs 50.3	0.63 (0.45-0.89)	0.009	>200	27	29	63.3 vs 47.8	0.89 (0.38-2.11)	0.798	0.51
≤240	175	186	58.3 vs 51.5	0.67 (0.48-0.94)	0.02	>240	23	20	56.3 vs 32.2	0.60 (0.23-1.56)	0.294	0.75

Supplementary Table 10 (A): Cut point analysis to assess predictive effect of membrane EGFR H-score (continued)

Overall survival (OS)

**Low EGFR (membrane) H-score**						**High EGFR (membrane) H-score**						
**Cut point**	**NCRT (n)**	**CRT (n)**	**4 year OS (months)**	**HR (95% CI) (NCRT vs CRT)**	***P****	**Cut point**	**NCRT (n)**	**CRT (n)**	**4 year OS (months)**	**HR (95% CI) (NCRT vs CRT)**	***P****	***P* (Interaction)**
≤10	29	35	56.1 vs 45.9	0.68 (0.33-1.41)	0.3	>10	169	171	46.0 vs 41.8	0.81 (0.59-1.09)	0.164	0.59
≤40	53	59	55.4 vs 41.9	0.73 (0.42-1.26)	0.264	>40	145	147	44.5 vs 43.3	0.80 (0.58-1.12)	0.196	0.72
≤60	74	73	53.0 vs 44.7	0.74 (0.45-1.19)	0.21	>60	124	133	44.2 vs 41.8	0.81 (0.57-1.15)	0.244	0.71
≤80	92	85	49.7 vs 43.6	0.80 (0.518-1.2)	0.295	>80	106	121	45.7 vs 42.4	0.77 (0.53-1.13)	0.184	0.97
**≤100**	**101**	**103**	**47.3 vs 44.8**	**0.89 (0.59-1.32)**	**0.548**	**>100**	**97**	**103**	**48.2 vs 40.7**	**0.69 (0.46-1.03)**	**0.071**	**0.40**
≤120	120	119	45.4 vs 41.0	0.87 (0.61-1.25)	0.456	>120	78	87	52.4 vs 44.4	0.65 (0.41-1.03)	0.069	0.32
≤140	133	141	44.4 vs 42.8	0.90 (0.64-1.26)	0.535	>140	65	65	55.4 vs 41.4	0.57 (0.34-0.96)	0.034	0.14
≤160	156	158	47.0 vs 42.3	0.80 (0.58-1.09)	0.158	>160	42	48	51.8 vs 42.8	0.72 (0.39-1.33)	0.295	0.77
≤180	166	168	47.0 vs 42.3	0.79 (0.58-1.07)	0.124	>180	32	38	52.6 vs 42.7	0.74 (0.36-1.51)	0.409	0.89
≤200	171	177	47.1 vs 42.9	0.80 (0.59-1.08)	0.138	>200	27	29	52.6 vs 46.4	0.68 (0.31-1.49)	0.335	0.72
≤240	175	186	47.3 vs 42.6	0.81 (0.60-1.09)	0.166	>240	23	20	51.9 vs 37.5	0.57 (0.24-1.35)	0.199	0.44

*Univariate Cox regression analysis. Results at median cut point are highlighted in bold. CRT=cisplatin-radiation alone; NCRT=nimotuzumab plus cisplatin-radiation; HR=hazard ratio; CI=confidence interval.

Supplementary Table 10 (B): Cut point analysis to assess predictive effect of cytoplasmic EGFR H-score

Progression free survival (PFS)

Low EGFR H-score						High EGFR H-score						
**Cut point**	**NCRT (n)**	**CRT (n)**	**4 year PFS (months)**	**HR (95% CI) (NCRT vs CRT)**	***P****	**Cut point**	**NCRT (n)**	**CRT (n)**	**4 year PFS (months)**	**HR (95% CI) (NCRT vs CRT)**	***P****	***P* (Interaction)**
≤20	14	15	76.9 vs 40.0	0.77 (0.26-2.28)	0.63	>20	184	191	50.4 vs 42.0	0.78 (0.59-1.05)	0.102	0.902
≤40	28	30	60.4 vs 40.2	0.72 (0.34-1.52)	0.389	>40	170	176	51.2 vs 42.3	0.80 (0.59-1.08)	0.14	0.715
≤60	41	47	56.1 vs 43.6	0.89 (0.48-1.67)	0.725	>60	157	159	51.8 vs 41.6	0.75 (0.55-1.03)	0.078	0.688
≤80	67	74	51.4 vs 41.3	0.84 (0.52-1.34)	0.46	>80	131	132	53.5 vs 42.1	0.74 (0.53-1.06)	0.097	0.706
≤100	77	83	54.8 vs 43.0	0.77 (0.49-1.21)	0.253	>100	121	123	51.3 vs 41.0	0.78 (0.54-1.11)	0.166	0.971
≤120	91	94	55.2 vs 44.4	0.74 (0.49-1.13)	0.166	>120	107	112	50.3 vs 39.9	0.80 (0.55-1.17)	0.257	0.765
**≤140**	**108**	**121**	**51.7 vs 45.9**	**0.79 (0.54-1.14)**	**0.204**	**>140**	**90**	**85**	**53.9 vs 35.7**	**0.76 (0.49-1.18)**	**0.228**	**0.942**
≤160	138	155	52.3 vs 46.9	0.82 (0.59-1.15)	0.254	>160	60	51	53.4 vs 27.4	0.65 (0.39-1.10)	0.111	0.477
≤180	167	190	50.8 vs 41.2	0.75 (0.56-1.02)	0.066	>180	31	16	64.0 vs 51.4	1.12 (0.43-2.90)	0.812	0.44

Loco-regional control (LRC)

**Cut point**	**NCRT (n)**	**CRT (n)**	**4 year LRC (months)**	**HR (95% CI) (NCRT vs CRT)**	***P****	**Cut point**	**NCRT (n)**	**CRT (n)**	**4 year LRC (months)**	**HR (95% CI) (NCRT vs CRT)**	***P****	***P* (Interaction)**
≤20	14	15	76.9 vs 40.0	0.26 (0.07-0.97)	0.44	>20	184	191	57.7 vs 50.2	0.71 (0.51-0.99)	0.041	0.146
≤40	28	30	71.4 vs 40.2	0.36 (0.15-0.87)	0.023	>40	170	176	57.3 vs 51.1	0.73 (0.52-1.03)	0.07	0.147
≤60	41	47	63.7 vs 48.4	0.58 (0.29-1.15)	0.121	>60	157	159	58.4 vs 49.8	0.68 (0.48-0.98)	0.039	0.68
≤80	67	74	56.1 vs 47.4	0.69 (0.42-1.15)	0.154	>80	131	132	61.5 vs 50.4	0.64 (0.42-0.96)	0.033	0.792
≤100	77	83	59.0 vs 51.0	0.71 (0.43-1.16)	0.166	>100	121	123	59.9 vs 48.2	0.62 (0.41-0.95)	0.028	0.707
≤120	91	94	58.5 vs 51.4	0.75 (0.47-1.17)	0.204	>120	107	112	60.0 vs 47.8	0.58 (0.37-0.92)	0.019	0.45
**≤140**	**108**	**121**	**57.0 vs 52.6**	**0.79 (0.52-1.18)**	**0.248**	**>140**	**90**	**85**	**62.6 vs 44.1**	**0.51 (0.31-0.85)**	**0.01**	**0.2**
≤160	138	155	58.4 vs 54.1	0.77 (0.53-1.13)	0.18	>160	60	51	62.3 vs 34.4	0.40 (0.22-0.75)	0.004	0.088
≤180	167	190	57.8 vs 48.5	0.66 (0.48-0.93)	0.017	>180	31	16	69.9 vs 61.2	0.68 (0.22-2.08)	0.498	0.93

Supplementary Table 10 (B): Cut point analysis to assess predictive effect of cytoplasmic EGFR H-score (continued)

Overall survival (OS)

Low EGFR H-score						High EGFR H-score						
**Cut point**	**NCRT (n)**	**CRT (n)**	**4 year OS (months)**	**HR (95% CI) (NCRT vs CRT)**	***P****	**Cut point**	**NCRT (n)**	**CRT (n)**	**4 year OS (months)**	**HR (95% CI) (NCRT vs CRT)**	***P****	***P* (Interaction)**
≤20	14	15	57.1 vs 52.5	0.77 (0.26-2.28)	0.63	>20	184	191	46.5 vs 41.4	0.78 (0.59-1.05)	0.102	0.902
≤40	28	30	55.2 vs 43.2	0.72 (0.34-1.52)	0.389	>40	170	176	46.1 vs 42.4	0.80 (0.59-1.08)	0.14	0.715
≤60	41	47	52.0 vs 50.1	0.89 (0.48-1.67)	0.725	>60	157	159	46.6 vs 40.4	0.75 (0.55-1.03)	0.078	0.688
≤80	67	74	50.6 vs 43.4	0.84 (0.52-1.35)	0.46	>80	131	132	46.8 vs 41.6	0.74 (0.53-1.06)	0.097	0.706
≤100	77	83	51.8 vs 47.4	0.77 (0.49-1.21)	0.253	>100	121	123	45.6 vs 40.5	0.78 (0.54-1.11)	0.166	0.971
≤120	91	94	46.9 vs 40.5	0.74 (0.49-1.13)	0.166	>120	107	112	52.4 vs 43.9	0.80 (0.55-1.17)	0.257	0.765
**≤140**	**108**	**121**	**49.7 vs 40.8**	**0.79 (0.54-1.14)**	**0.204**	**>140**	**90**	**85**	**45.7 vs 45.9**	**0.76 (0.49-1.18)**	**0.228**	**0.942**
≤160	138	155	50.4 vs 43.5	0.82 (0.59-1.15)	0.254	>160	60	51	43.0 vs 38.5	0.65 (0.39-1.10)	0.111	0.477
≤180	167	190	50.0 vs 41.4	0.75 (0.56-1.02)	0.066	>180	31	16	35.2 vs 55.1	1.12 (0.43-2.90)	0.812	0.44

*Univariate Cox regression analysis. Results at median cut point are highlighted in bold. CRT=cisplatin-radiation alone; NCRT=nimotuzumab plus cisplatin-radiation; HR=hazard ratio; CI=confidence interval.

Supplementary Table 11: Combined analysis of HIF1α and EGFR (membrane) for its predictive significance

**Biomarker combination**	**Events/n (NCRT)**	**Events/n (CRT)**	**4 YR survival (months)**	**HR (95% CI)**	***P****	***P* (interaction)**
**Progression free survival (PFS)**						
Both low	22/51	30/64	48.2 vs 46.8	0.91 (0.53-1.58)	0.747	0.327
HIF1α high-EGFR low	20/48	21/34	56.8 vs 34.3	0.52 (0.28-0.96)	**0.036**	
HIF1α low-EGFR high	16/41	21/44	50.1 vs 46.4	0.76 (0.40-1.47)	0.418	
Both high	22/53	33/57	53.1 vs 36.6	0.57 (0.33-0.98)	**0.04**	
**Loco-regional control (LRC)**						
Both low	15/51	23/64	63.9 vs 58.3	0.82 (0.43-1.57)	0.546	0.418
HIF1α high-EGFR low	18/48	18/34	60.1 vs 42.0	0.57 (0.29-1.09)	0.088	
HIF1α low-EGFR high	13/41	16/44	55.5 vs 53.6	0.78 (0.38-1.63)	0.51	
Both high	19/53	30/57	61.0 vs 40.6	0.54 (0.30-0.96)	**0.036**	
**Overall survival (OS)**						
Both low	25/51	30/64	42.6 vs 47.7	1.07 (0.63-1.82)	0.797	0.127
HIF1α high-EGFR low	21/48	21/34	50.4 vs 30.9	0.60 (0.33-1.10)	0.097	
HIF1α low-EGFR high	22/41	17/44	40.0 vs 54.6	1.23 (0.65-2.32)	0.518	
Both high	21/53	36/57	53.5 vs 32.6	0.51 (0.29-0.87)	**0.013**	

*Univariate Cox regression analysis. Respective median H-score values were used as the cut point for categorization [HIF1α=90; EGFR=100]. NCRT= nimotuzumab plus cisplatin-radiation; CRT= cisplatin-radiation alone; HR=hazard ratio; CI= confidence interval.

SUPPLEMENTARY FIGURES

(A)
(B)

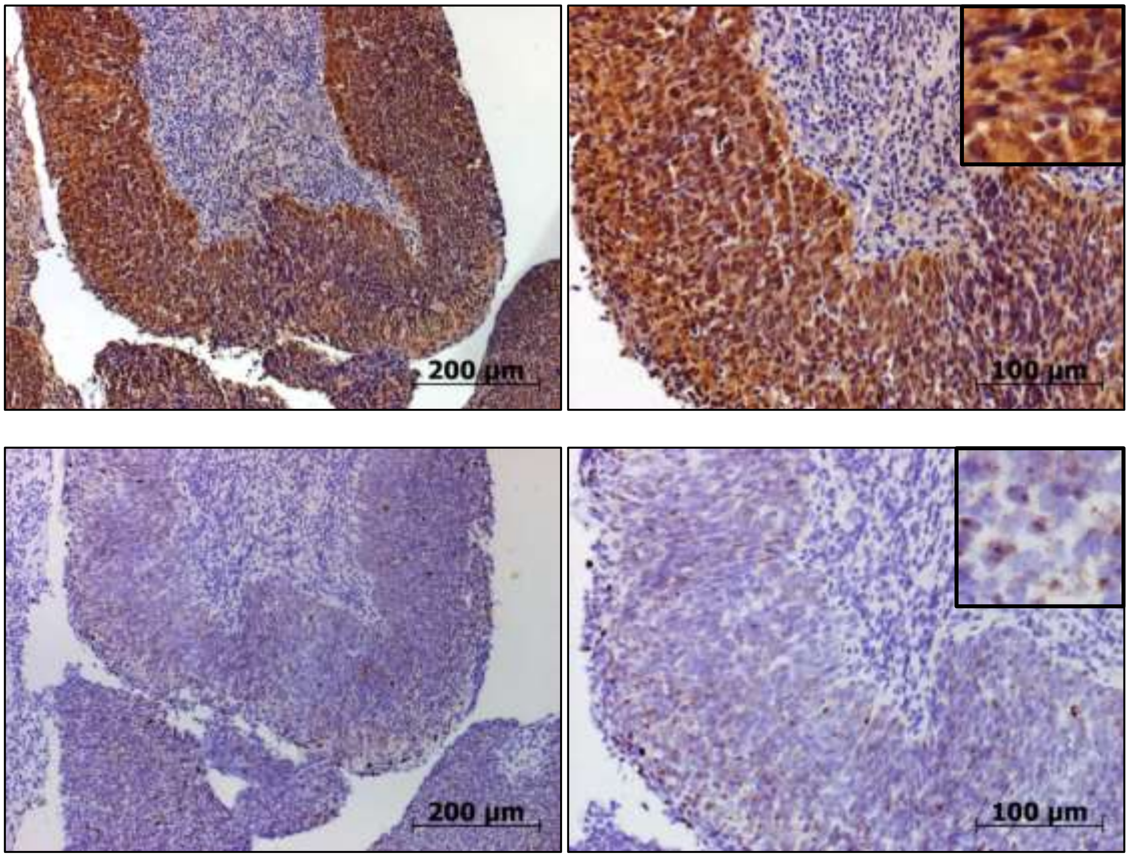

Supplementary Figure 1. Human papilloma virus (HPV) positive HNSCC. (A) Representative immunohistochemistry image showing strong, diffuse expression of p16 in nucleus and cytoplasm. (B) RNA-*insitu* hybridization result showing presence of high risk HPV E6/E7 mRNA (seen as brown puncta in the tumor cell nuclei) in the same field.

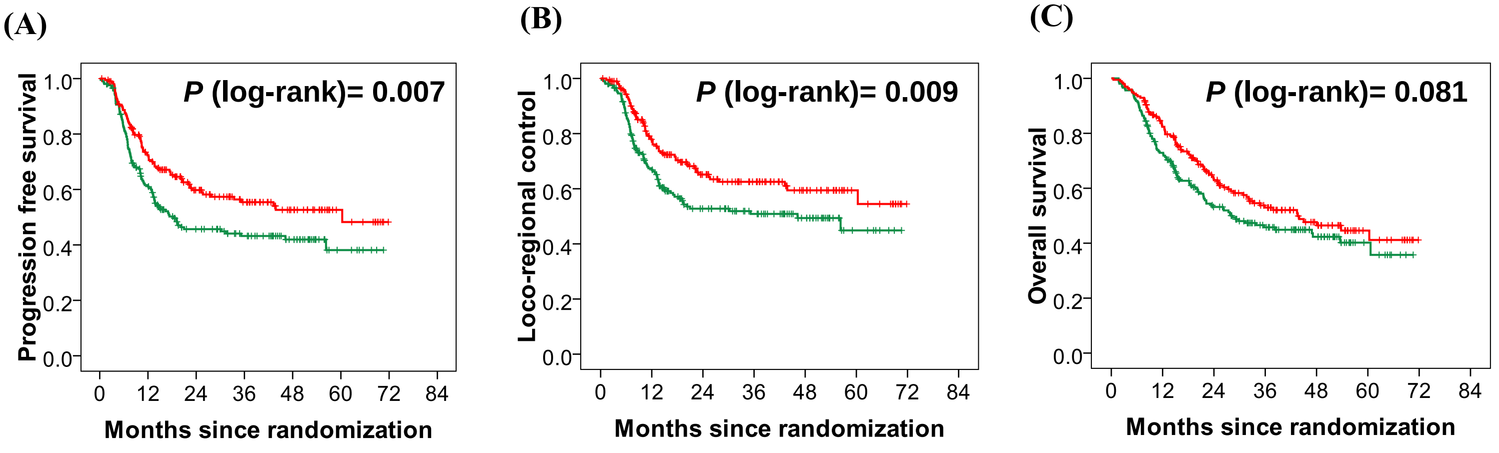

	CRT	NCRT
Events/n	108/206	80/198
4 Year PFS	41.9 vs 52.6 months	
HR (95%CI)	0.67 (0.51-0.90)	

	CRT	NCRT
Events/n	90/206	65/198
4 Year LRC	49.4 vs 59.4 months	
HR (95%CI)	0.66 (0.48-0.90)	

	CRT	NCRT
Events/n	106/206	89/198
4 Year OS	42.3 vs 47.7 months	
HR (95%CI)	0.78 (0.59-1.03)	

Supplementary Figure 2. Kaplan-Meier curves. (A) progression free survival (PFS), (B) loco-regional control (LRC) and (C) overall survival (OS) in biomarker subgroup (n=404).

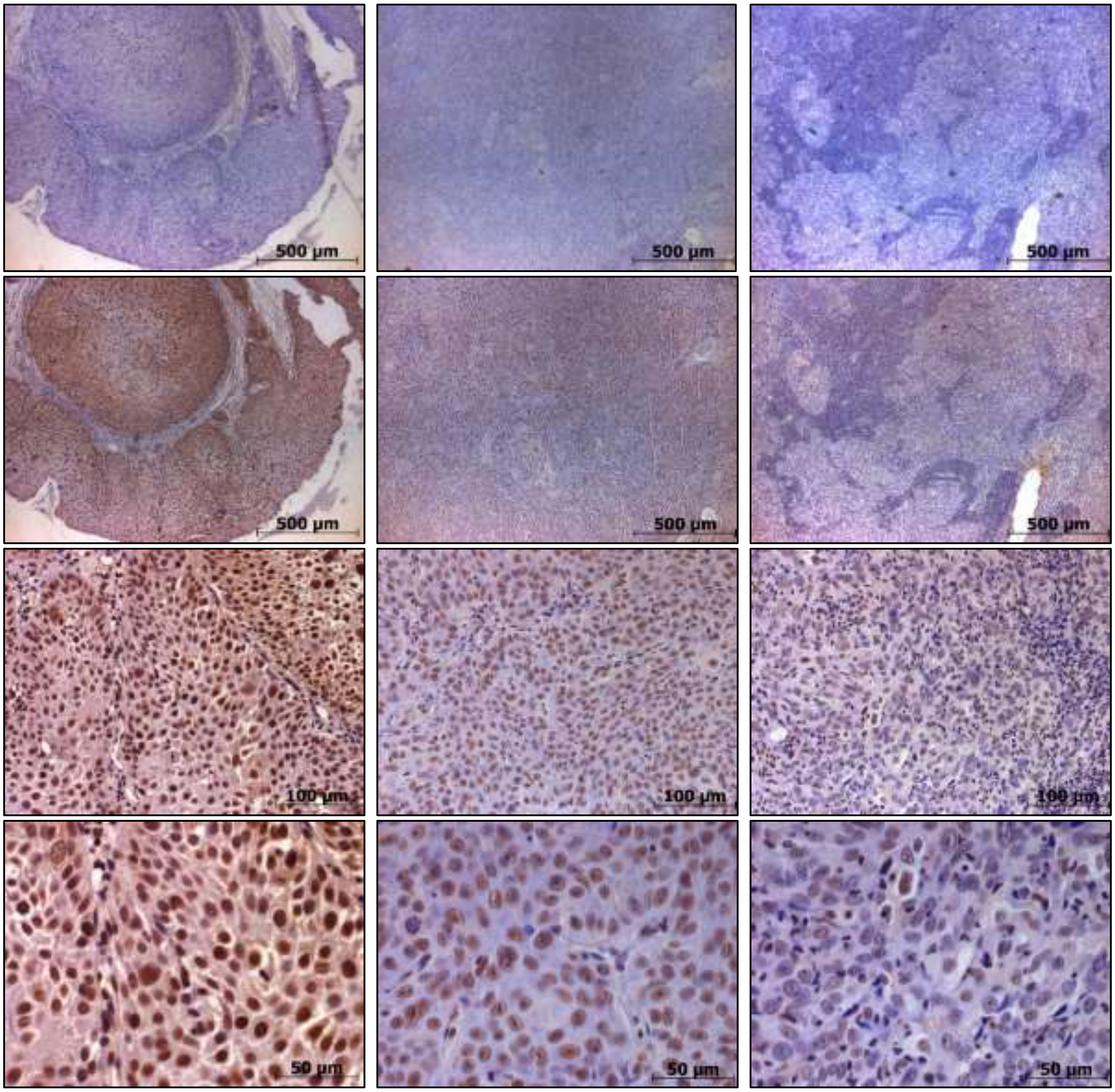

High (H-score=270) **High (H-score=180)** **Low (H-score=70)**

Supplementary Figure 3A. Representative immunohistochemistry (IHC) staining results. High and low nuclear staining of HIF1α, uppermost panel shows respective isotype control.

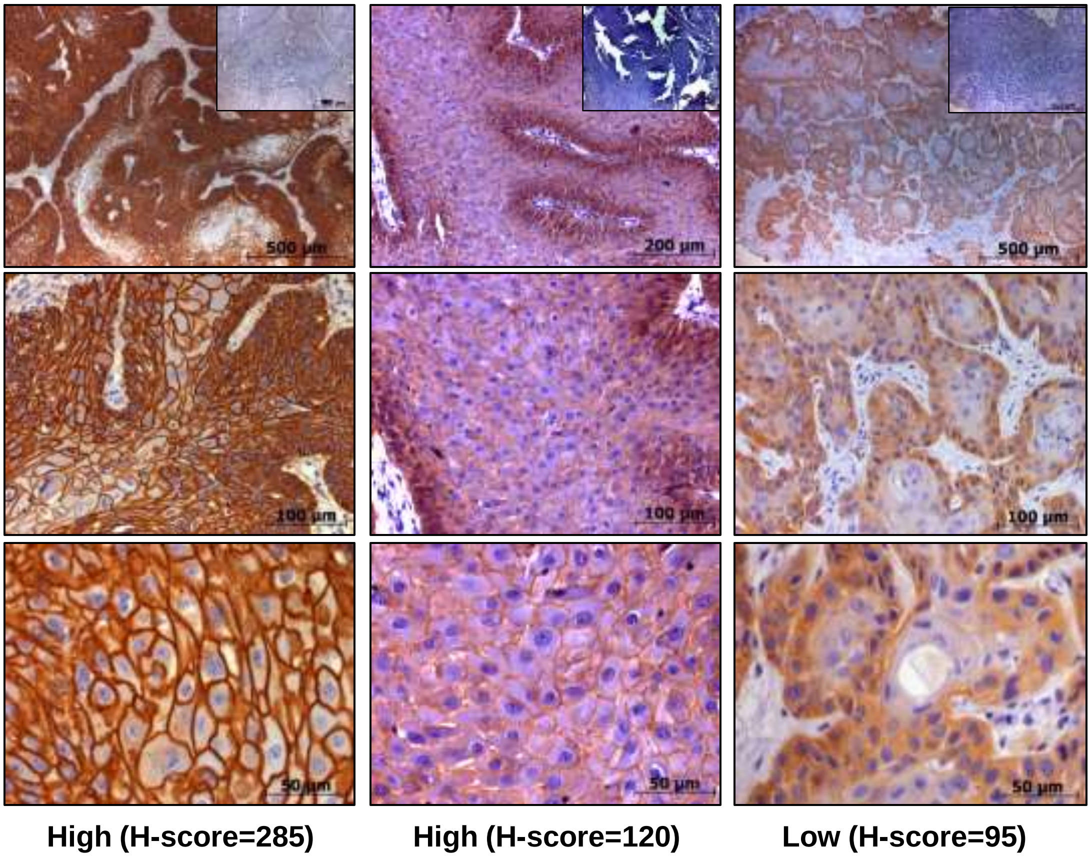

Supplementary Figure 3B. Representative immunohistochemistry (IHC) staining results. High and low membranous staining of EGFR, inset shows respective isotype control.

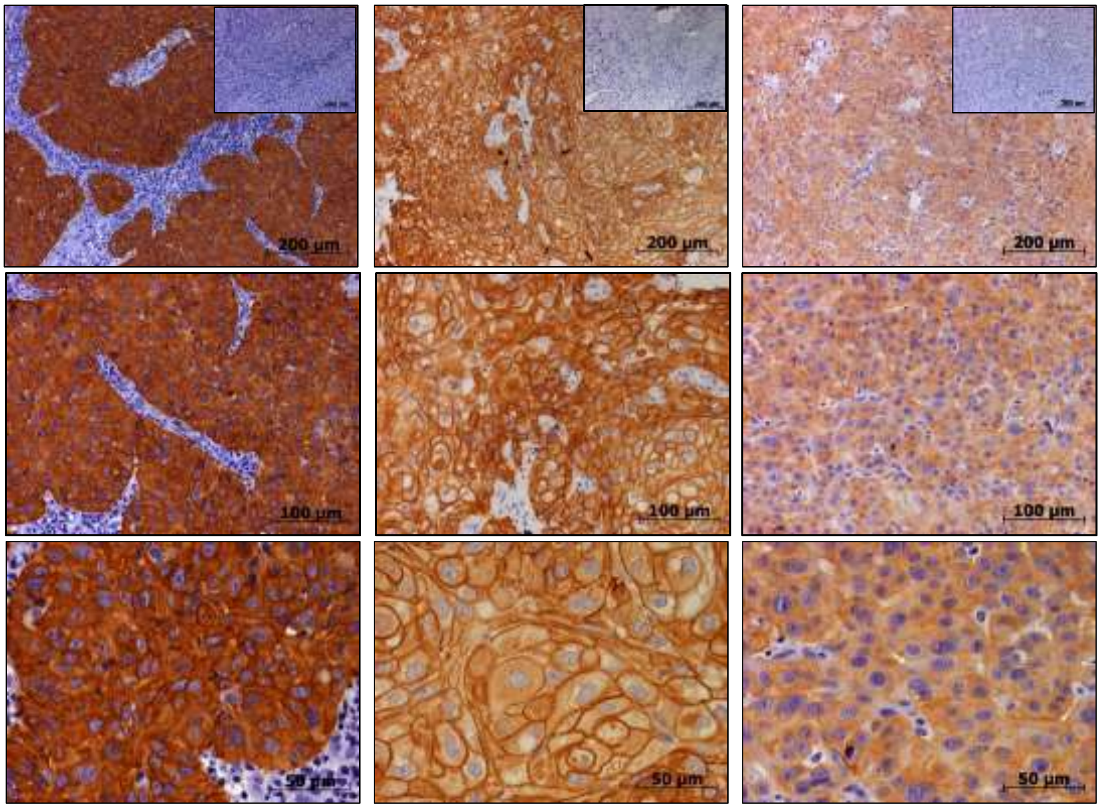

High (H-score=300) High (H-score=180) Low (H-score=95)

Supplementary Figure 3C. Representative immunohistochemistry (IHC) staining results. High and low cytoplasmic staining of EGFR, inset shows respective isotype control.

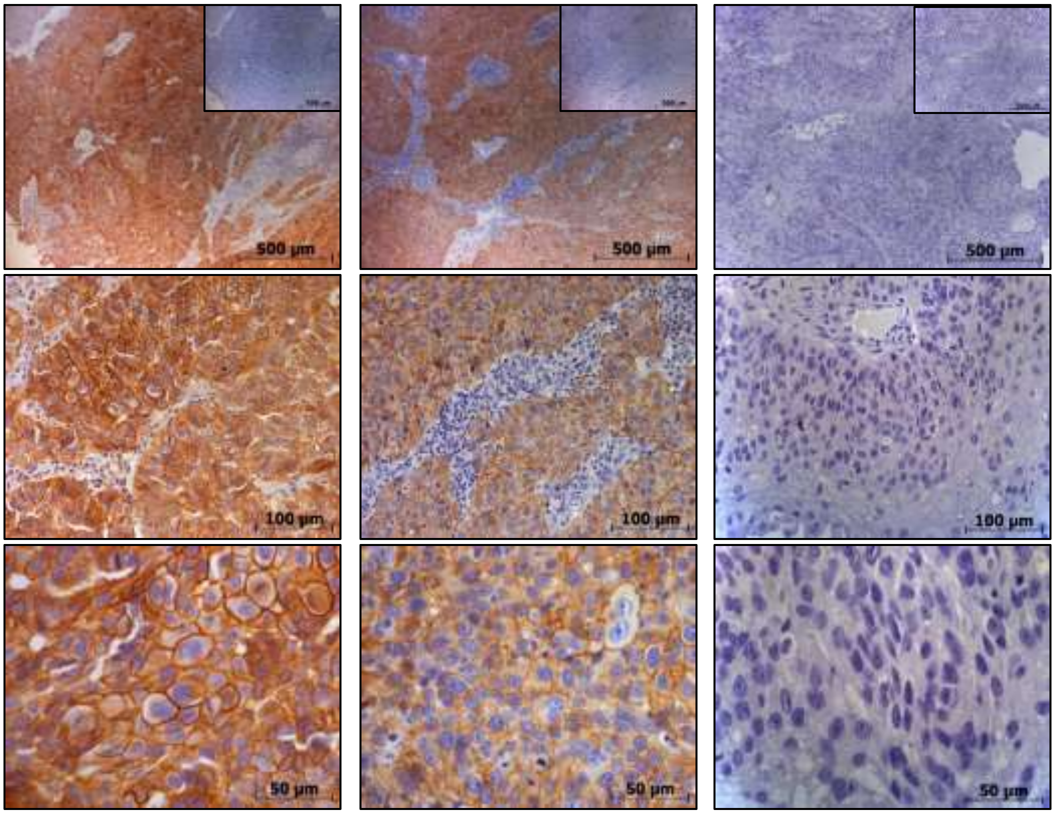

Positive (H-score=180) **Positive (H-score=150)** **Negative (H-score=0)**

Supplementary Figure 4A. Representative immunohistochemistry (IHC) staining results. Positive and negative membrane staining of pEGFRY1068, inset shows respective isotype control.

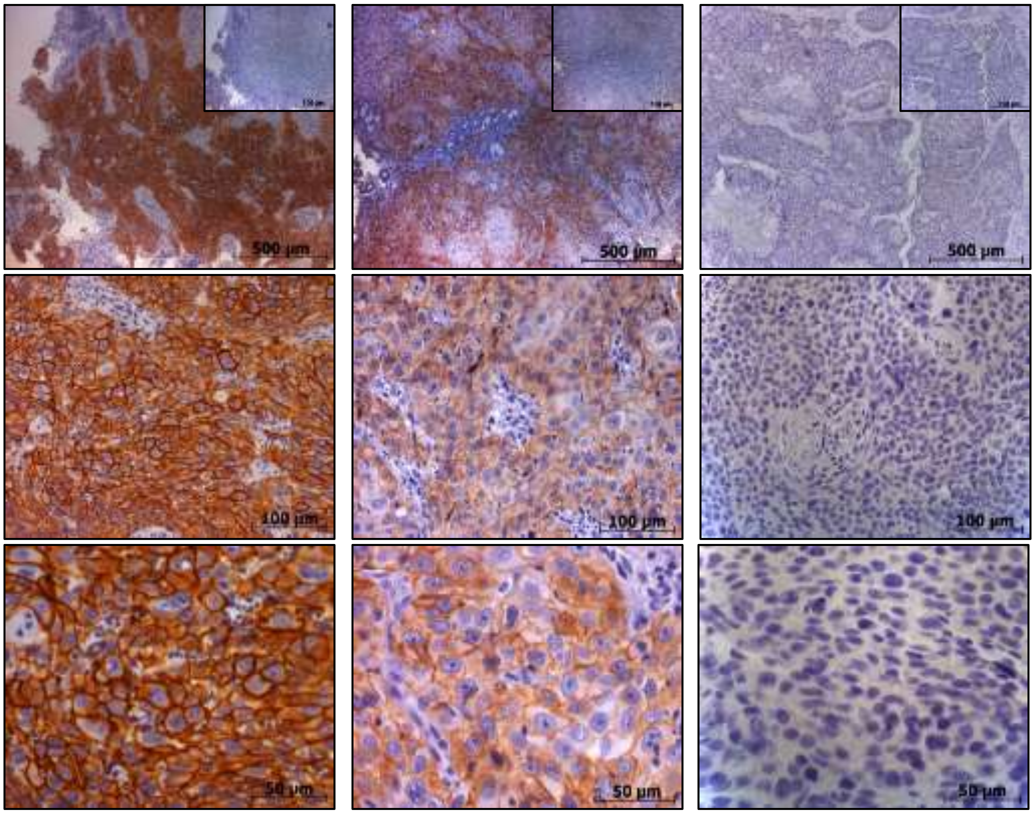

Positive (H-score=225) **Positive (H-score=160)** **Negative (H-score=0)**

Supplementary Figure 4B. Representative immunohistochemistry (IHC) staining results. Positive and negative membrane staining of pEGFRY1173, inset shows respective isotype control.

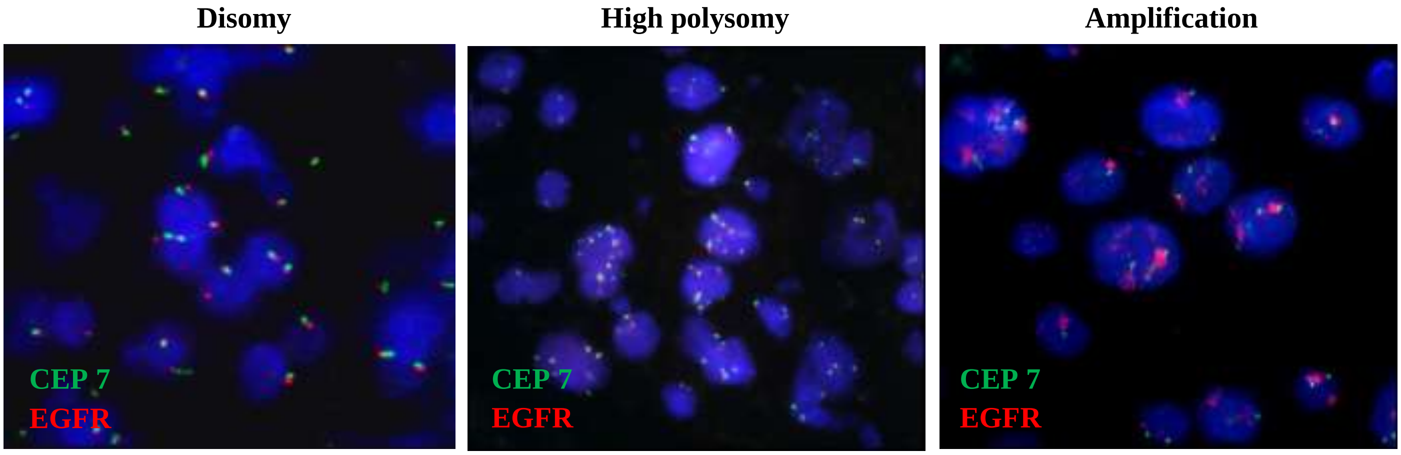

Supplementary Figure 4C. Representative fluorescence in situ hybridization (FISH) results. EGFR FISH results showing tumor cells with disomy [≤2 copies in >90% of cells]; high polysomy [≥4 copies in ≥40% of cells] and gene amplification seen as large cluster of EGFR signals.

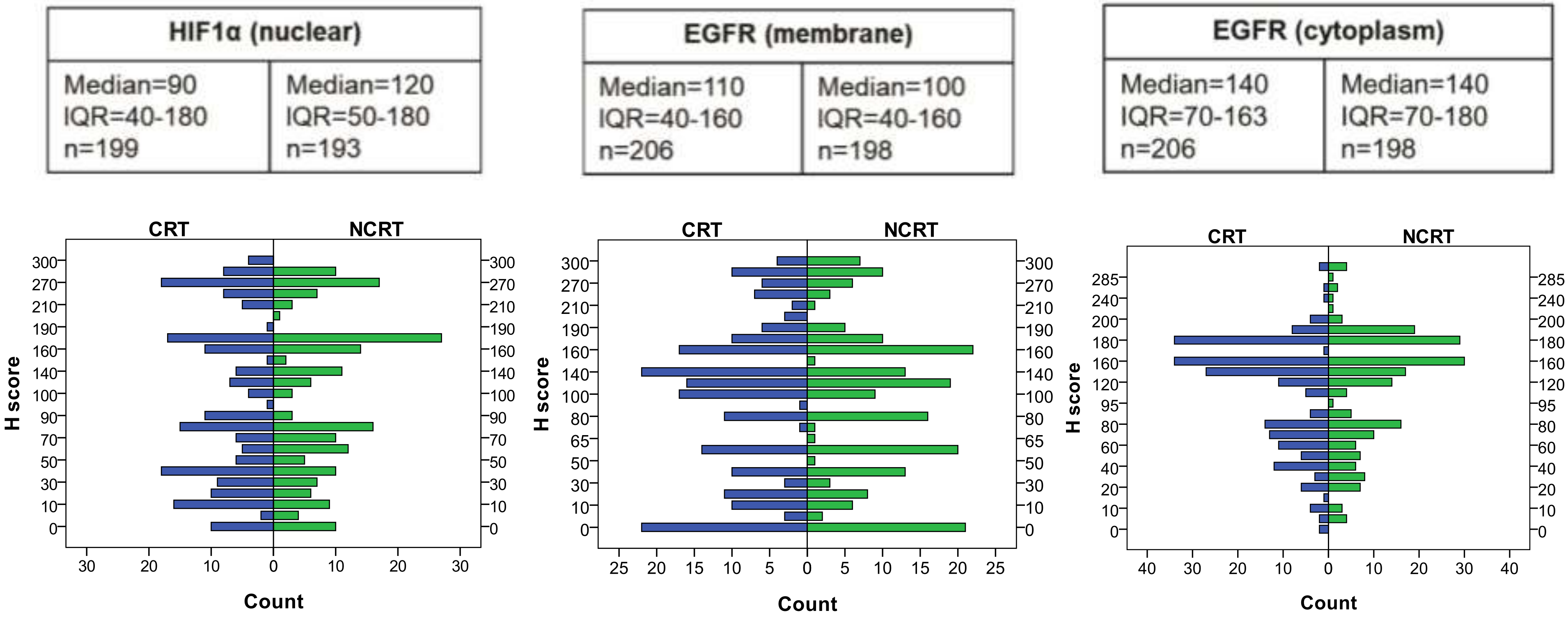

Supplementary Figure 5 (A-C). Histograms showing frequency distribution of different protein biomarkers across treatment groups. (A) HIF1α, (B) EGFR (membrane), (C) EGFR (cytoplasm); CRT=cisplatin-radiation; NCRT=nimotuzumab plus cisplatin-radiation; IQR= inter quartile range.

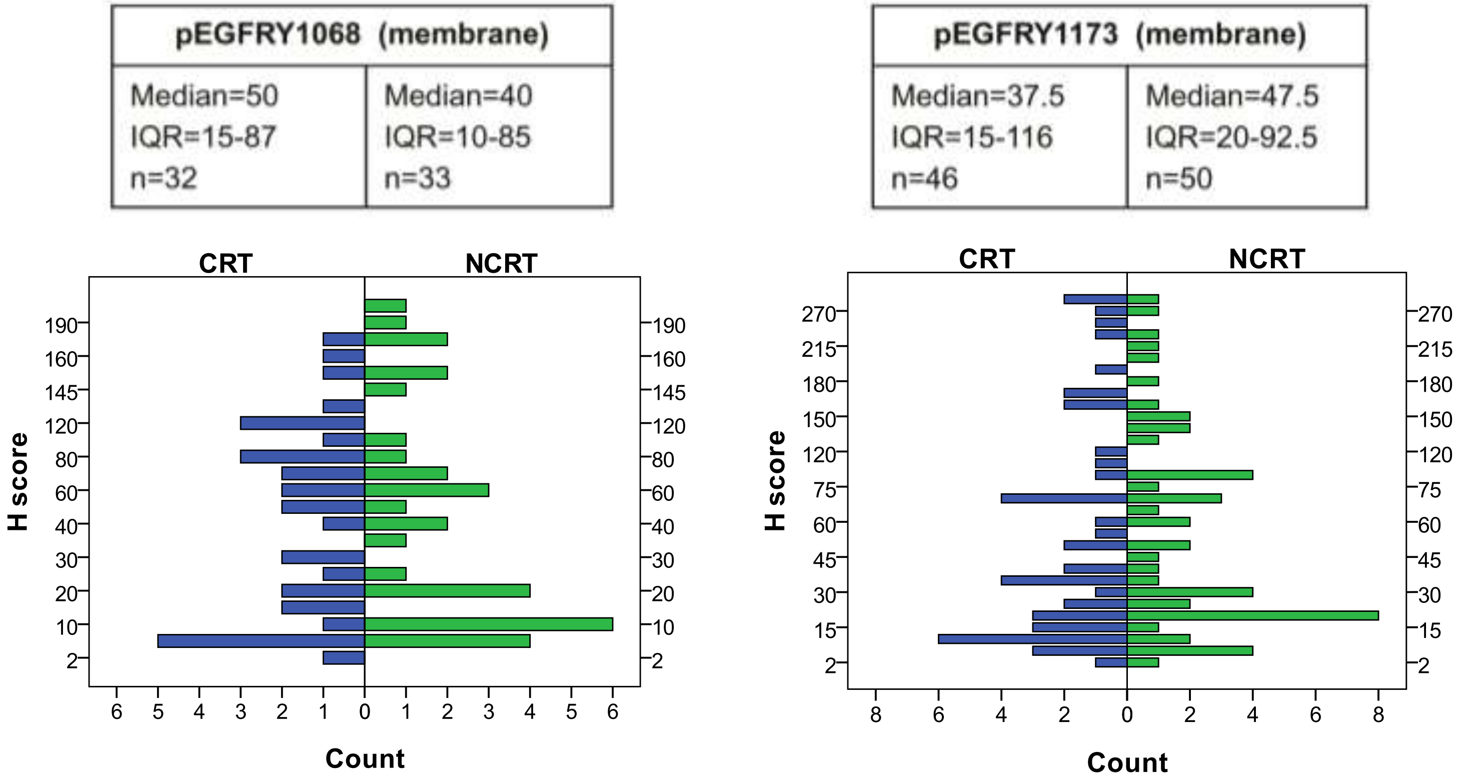

Supplementary Figure 5 (D-E). Histograms showing frequency distribution of different protein biomarkers across treatment groups. (D) pEGFRY1068 (membrane) and (E) pEGFRY1173 (membrane); CRT=cisplatin-radiation; NCRT=nimotuzumab plus cisplatin-radiation; IQR= inter quartile range.

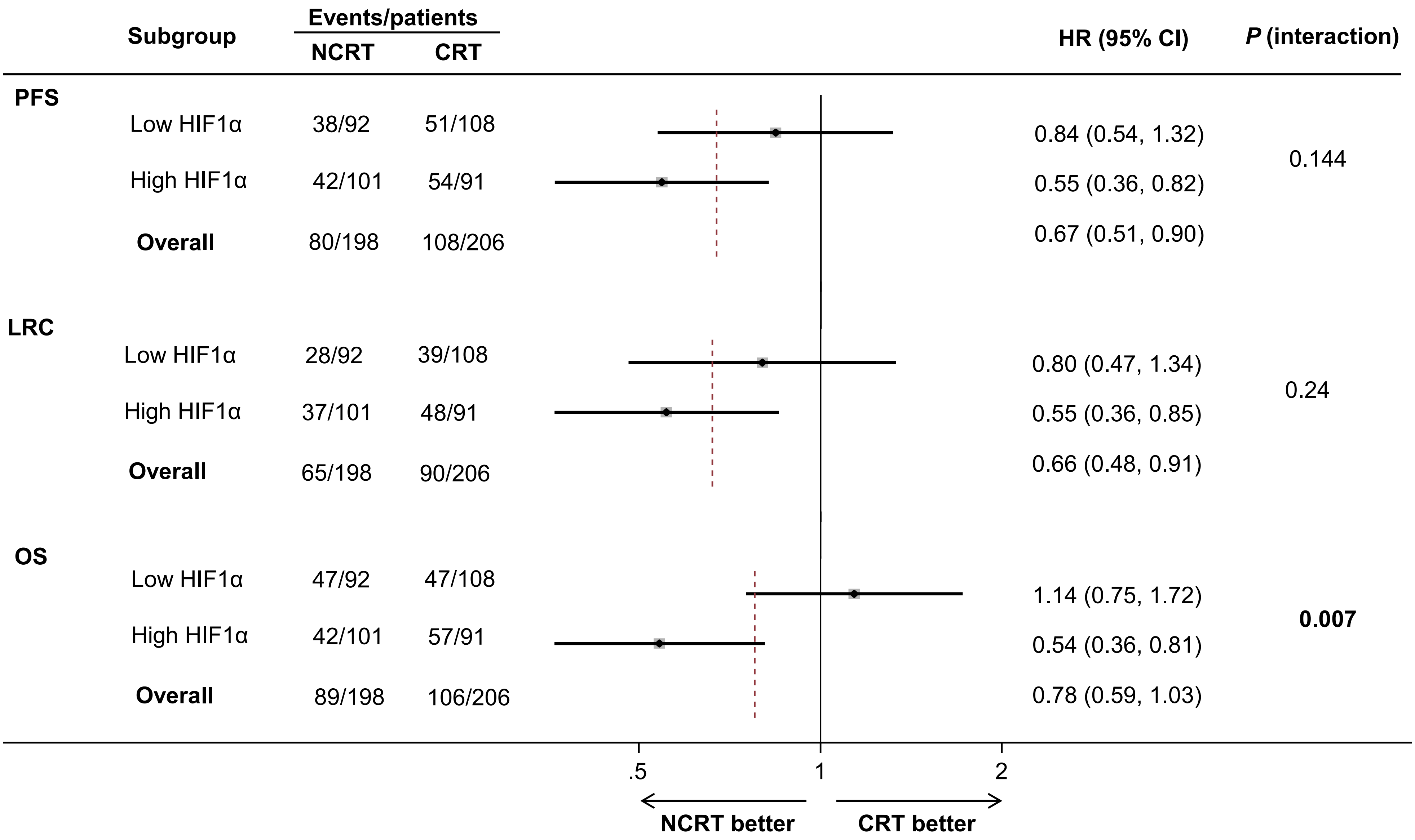

Supplementary Figure 6. Forest plots showing bootstrap resampling results for PFS (A), LRC (B) and OS (C) by HIF1α expression subgroups. The interaction P value is based on a two-sided test of interaction between treatment and HIF1α expression status in the Cox proportional hazards model. A hazard ratio (HR) of less than 1 indicates a benefit with the addition of nimotuzumab; CI=confidence interval; dotted line represents the respective hazard ratio for the overall study population.

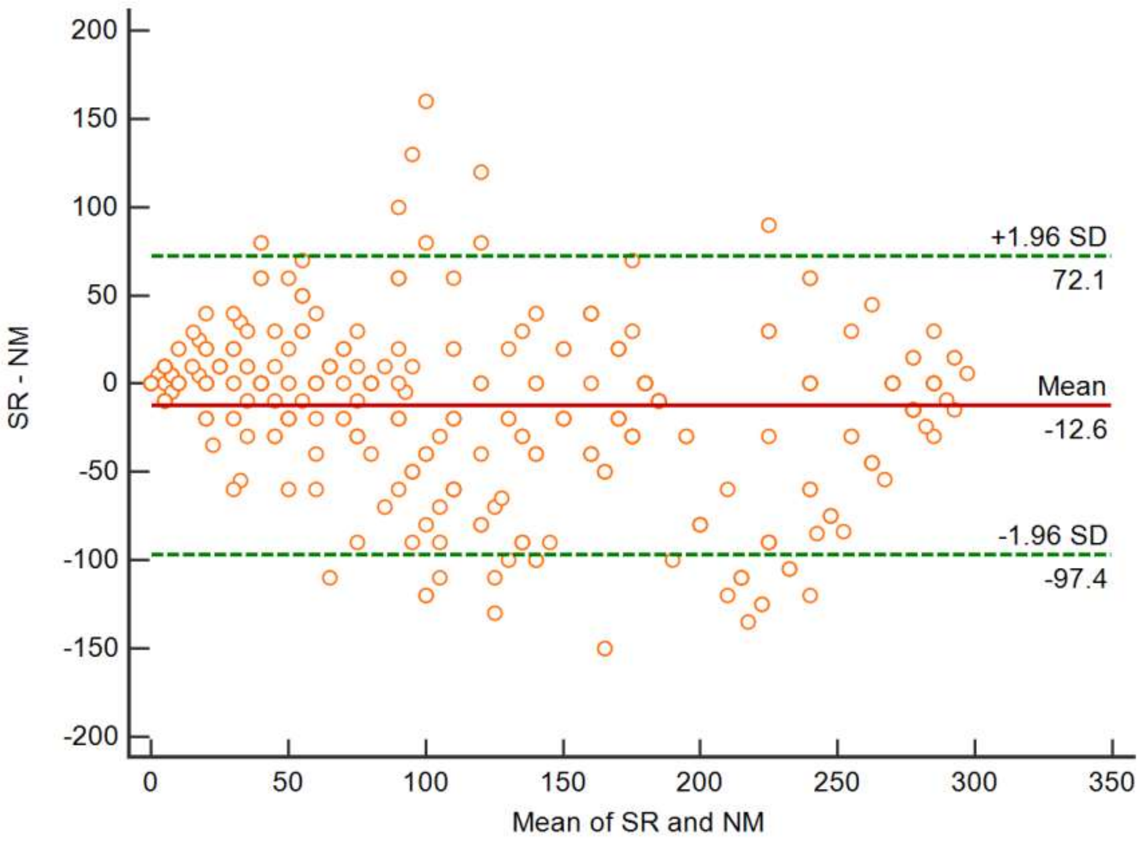

Supplementary Figure 7. Bland-Altman plot showing agreement between HIF1α scoring of two pathologists. The vertical axis indicates the difference between the two pathologist's (SR and NM) H-score and the horizontal axis is the average of the two. The solid red line represents the mean value and the dashed green show ±1.96 SD.
